# EU Wide Disaggregated CAPRI Model Data: Crops, Livestock, Nitrogen In- and Outputs (Timeseries 2000-2018)

**DOI:** 10.1038/s41597-026-06919-8

**Published:** 2026-03-05

**Authors:** Renate Koeble, Adrian Leip, Markus Kempen, Jan-Erik Petersen, Oscar Gomez, Debbora Leip, Rui Catarino, Maria Bielza, Franz Weiss, Xavier Rotllan-Puig, Maria Luisa Paracchini, Linda See, Marijn van der Velde

**Affiliations:** 1 ARHS Developments S.A., 4370 Belvaux, Luxembourg; 2 European Commission, DG Research and Innovation, 1049 Brussels, Belgium; 3 EuroCARE, 53123 Bonn, Germany; 4 European Environment Agency, 1050 Copenhagen, Denmark; 5 European Commission, Eurostat, 2721 Kirchberg, Luxembourg; 6 Potsdam Institute for Climate Impact Research - Member of the Leibniz Association, 14412 Potsdam, Germany; 7 Department of Agricultural Economics, Humboldt-Universität zu Berlin, 10115 Berlin, Germany; 8 European Commission, Joint Research Centre (JRC), Ispra, Italy; 9 Independent researcher (Seidor), 21027 Ispra, VA Italy; 10 ASTER-Projects, 08694 Guardiola de Berguedà, Barcelona, Spain; 11 International Institute for Applied Systems Analysis, 2361 Laxenburg, Austria

**Keywords:** Agroecology, Environmental impact

## Abstract

The impacts of agricultural, environmental and climate change policies in Europe are investigated using the CAPRI (Common Agricultural Policy Regionalised Impact) model. Although the model runs at a regional level, primarily NUTS2, the underlying data have been spatially disaggregated to finer scale units called ‘Farm Structure Units’. In this study we present these spatially disaggregated layers of agri-environmental variables for a time series from 2000 to 2018. This harmonized and spatially consistent data set includes crop types (area, yield), livestock types (totals and densities) and various parameters associated with nitrogen application and losses. These data can be used to calculate nitrogen budgets as well as providing inputs to biogeochemical or land management models.

## Background & Summary

Official statistics from surveys and censuses of population and agriculture are often released as aggregated data by administrative zone to comply with legal confidentiality requirements^1^. For example, Eurostat releases agricultural statistics on crop production and livestock numbers at NUTS2 level, an administrative zone two levels below the national level^2,3^. However, real-world agricultural and livestock systems are highly heterogeneous due to differences in soil types, climate conditions, access to water and markets, and management practices, and can, therefore, vary substantially at a much finer resolution than a NUTS2 region^4^. Downscaling aggregate data into finer-resolution grids allows analysts and policymakers to align the data with the scale of the decisions, targeting specific hotspots where they occur. Moreover, many environmental processes are highly non-linear, so if the analysis of environmental impacts is based on data at a relatively coarse scale such as NUTS2 regions, the conclusions can be misleading^5^. Identification of hotspots or the local excess of thresholds is often more meaningful than average values. Gridded, downscaled inputs are also required for agricultural, hydrological and economic models so that yields, water use, emissions and other environmental parameters can be simulated consistently across space. In addition, downscaled data enable scenario testing and undertaking sensitivity analyses, which improves transparency regarding where interventions may work and where uncertainties remain.

Different approaches have been used to downscale administrative level data to higher resolution gridded data. For example, downscaled crop statistics are available from the Spatial Production Allocation Model (SPAM) for 2000, 2010 and 2020 at a 10 km × 10 km resolution^6,7^, utilizing a cross-entropy approach to allocate the crops based on agricultural area, suitability layers, irrigation and administrative level crop statistics to ensure that national and subnational areas are maintained. Then using cropping intensity, potentially attainable yields and yield statistics, the area harvested, the yield and the production for 46 crops are calculated. In contrast, the MIRCA-OS project^8^ has produced global gridded irrigated and rainfed cropped area for 2000 to 2015 through a stepwise downscaling approach that sequentially allocates irrigated and rainfed areas for 23 crops.

In addition to crops, other studies have focused on downscaling livestock data. These include the Gridded Livestock of the World (GLW) for 2010 and 2015 at a 10 km × 10 km resolution^9^, a 1 km × 1 km resolution product for Europe^10^, and more recently an annual global time series from 2000 to 2022 at a 1 km × 1 km resolution^11^. The GLW provides two versions of its gridded products, one produced by equal distribution of livestock numbers across administrative units while the second version uses machine learning to allocate livestock based on land use, human demographics, topography, vegetation and climate. A similar approach was used by Parente *et al*.^11^ except that a more extensive set of environmental, socioeconomic and anthropogenic input parameters were used to produce a higher resolution time series. In contrast, the 1 km × 1 km European product^10^ produced probability maps of livestock in two ways: derived from expert-based suitability rules and using a statistical approach. Livestock numbers were then allocated to these probability layers based on a prioritization process.

Finally, nitrogen budget parameters have also been downscaled. Tian *et al*.^12,13^ published a global data set on the evolution of anthropogenic nitrogen input since 1860 at a resolution of 5 arc minutes. Similarly Batool *et al*.^14^ released a long-term time series dataset of nitrogen surplus for both agricultural and non-agricultural soils, also at 5 arc minutes resolution. Both approaches employ a downscaling process from the country level to the grid, guided by existing spatially explicit information - such as crop areas, crop production, non-agricultural land cover, manure nitrogen production and application, and atmospheric deposition- as well as statistical data predominantly at the country level, to construct the time series. Due to limitations in spatial and statistical information at the global scale, both datasets partially rely on shared data sources, such as the Hyde 3.2^15^ dataset for land use, Monfreda *et al*.^16^ for harvested crop area, and Zhang *et al*.^17^ for manure nitrogen production and application. However, the methods for spatially allocating nitrogen inputs differ. The work of Tian *et al*.^13^ focuses on the historical development of nitrogen inputs, distinguishing between different forms of nitrogen, including synthetic NH₄ and NO₃, manure-derived nitrogen, and atmospheric deposition of NOₓ and NOᵧ. In contrast, Batool *et al*.^14^ investigate the development of nitrogen surplus over the past 170 years, considering both agricultural and non-agricultural soils. This includes nitrogen inputs and outputs to land uses beyond agriculture, such as biological nitrogen fixation in forests or semi-natural ecosystems for example. Focusing on Europe, De Vries *et al*.^18^ calculated spatially explicit agricultural N inputs and associated N losses based on the INTEGRATOR model for so-called Nitrogen Calculation Units (NCUs). The 40000 NCUs in the area of the EU are clusters of 1 km^2^ pixels with identical soil, slope and altitude classes in a sub-national administrative region. Crop areas, yields, livestock distribution are downscaled from administrative level (country or region) to the NCU based on a set of proxies available at higher spatial resolution, expert judgement and other auxiliary data. Nitrogen input, output and loss terms are then calculated at NCU level.

Unlike previous downscaling studies that primarily focus on specific aspects of agricultural management - such as crop areas or livestock or fertilizer inputs, or nitrogen losses to the environment - this paper presents a comprehensive set of disaggregated data covering individual crop and livestock activities, yields at crop-type level as well as nitrogen inputs, outputs and losses to the environment. The results are available as 1 km × 1 km raster data sets for a time series 2000–2018 at the Zenodo repository (10.5281/zenodo.13992301)^19^.

Regional-level results from the CAPRI (Common Agricultural Policy Regionalised Impact) model form the basis for the disaggregation. CAPRI is a modelling framework designed for ex-ante evaluation of agricultural and trade policies, with a focus on the European Union, but also incorporating trade between 44 regions in the rest of the world in its market module^20,21^. In addition, the model is used for impact assessments of agricultural activities on the environment (e.g., the nitrogen balance, GHG emissions, soil erosion or soil carbon stock changes) at regional and country level^22–27^. Model results for 231 European CAPRI regions, primarily at the NUTS2 level (EU27 excluding Croatia, including UK), were disaggregated to 215,199 Farm Structure Units (FSUs). The FSUs are spatially explicit, with varying sizes, ranging from a minimum of 1 km × 1 km to a maximum of 10 km × 10 km, with an average size of about 11 km². They are defined by the intersection of a 10 km × 10 km grid, soil mapping units, boundaries of the CAPRI regions and ‘no-go’ areas, where non-agricultural land cover (such as urban, water, forest) prevails. In the following we describe the disaggregation procedure from the regional level to the FSU for the individual parameters. Additionally, we compare our results with other spatial and statistical datasets to provide users with an understanding of the quality and uncertainties inherent in the disaggregated dataset.

## Methods

**Fig. 1:**
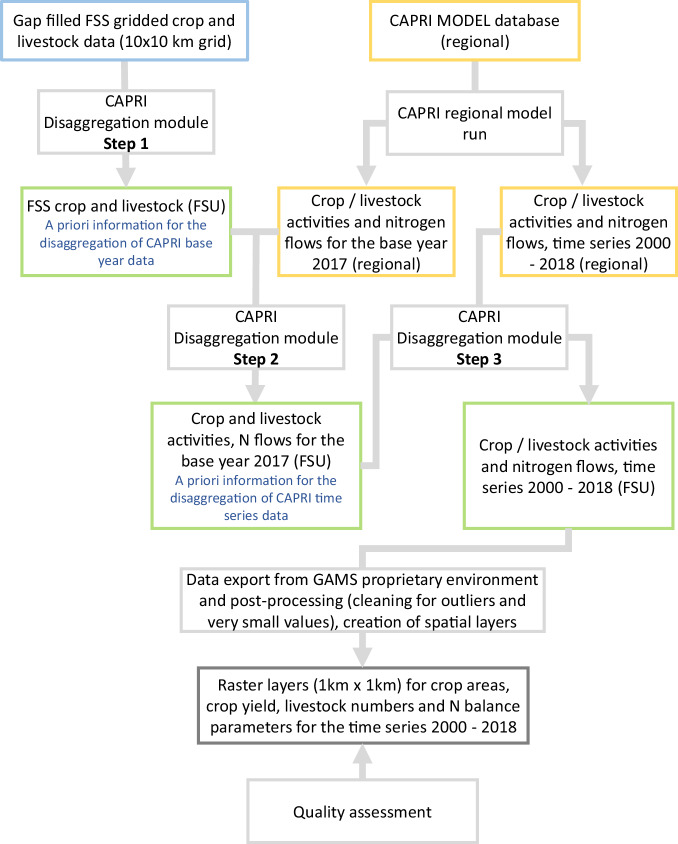
The methodological workflow to spatially disaggregate and evaluate CAPRI outputs. Text box border colors highlight the different spatial levels (blue: 10 km × 10 km grid, yellow: CAPRI NUTS regions, green: FSU).

The general methodology for the spatial disaggregation process for agricultural parameters - such as crop areas and yield, livestock numbers, nitrogen inputs/outputs and nitrogen losses - from the regional level of CAPRI, which primarily corresponds to the NUTS2 level of the EU Nomenclature of Territorial Units for Statistics, to the so-called Farm Structure Units (FSU) is provided in Fig. 1. The disaggregation procedure is implemented using the GAMS (General Algebraic Modelling System, https://www.gams.com/) programming language, on which the CAPRI model is built. GAMS is designed for solving complex mathematical optimization and equilibrium problems, which is essential in economic modelling. To create the base year (2017) as well as the time series data set at regional level and to run the disaggregation we used the CAPRI model trunk version (revision 10667). All the data necessary to execute the disaggregation described here has been incorporated into this version.

Gridded Farm Structure Survey (FSS) data for livestock numbers and crop areas for the year 2010 received from the Statistical Office of the European Union (Eurostat)^28^ provide the backbone for the disaggregation process (Fig. 1, top left). The data structure and pre-processing are described in a dedicated subsequent section.

In the **first step**, the gridded FSS data at a 10 km × 10 km resolution were disaggregated to FSUs, which are smaller sub-units of this grid described in the next section. Based on this *a priori* information, the CAPRI regional data for the base year 2017 was disaggregated to the FSU level in the **second step**. The disaggregation procedures differ for the different parameters (i.e., crops, livestock, irrigated areas, yield and nitrogen) and are detailed in the following sections. The data disaggregated for the base year itself finally becomes the prior information for disaggregating the CAPRI time series data for the years 2000 to 2018 in the **third step**. Starting from the base year 2017, we disaggregated all the parameters in annual steps back to the past or forward to later years. In this step, the spatial stability of the parameters was maximized, i.e., deviations from the actual pattern were allowed only if the model encounters certain situations, e.g., if a new crop was introduced in a specific region in a specific year. The sum of the disaggregated FSU level data in a region is consistent with the original CAPRI data in this region.

At the end, the results were exported from the GAMS proprietary format to the open source statistical environment R and cleaned for very small values and for outliers. Finally, spatial layers in.tif raster format were created. The 1 km^2^ pixel resolution of the rasters correspond to the minimum size of a FSU. The comparison of the results with other published data sets provides insights into the variation of the results due to different data inputs and methods (see the Technical Validation section).

### Definition and delineation of the farm structure units (FSUs)

The Farm Structure Units (FSU) represent an updated version of previously defined spatial units used for disaggregating CAPRI model data^29^. The main difference between FSUs and the previous spatial units is that each FSU falls completely within one 10 km × 10 km grid cell of the FSS data. An FSU has a minimum size of 1 km^2^ and is defined by the spatial intersection of the following input layers, which have been gridded to a 1 km^2^ pixel resolution beforehand:

- an INSPIRE compliant 10 km × 10 km grid available at the European Environment Agency (EEA)^30,31^, which provides the link to the gridded FSS crop and livestock data for 2010.
- borders of NUTS2 regions as of 2016^2^ and CAPRI NUTS regions if the latter do not coincide with the delineation of NUTS2 regions from Eurostat, which are collectively referred to as ‘regions’ from this point onwards.
- soil mapping units from the Food Agriculture Organization (FAO) dataset ‘Harmonized World Soil Database’^32^.
- ‘no-go areas’ which are defined as 1 km² grid cells where certain land cover classes, specifically ‘bare soils’, ‘glaciers’, ‘water bodies’, and ‘urban areas’, occupy more than 90% of the cell’s area. We assume that these land cover classes will be rather persistent in the longer run and no conversion to agricultural land is expected in the near future. The respective grid cells are delineated based on the EEA ‘CORINE land cover map 2018^33^ at 100 m resolution. A comparison of CORINE land cover data from 2012 and 2018, conducted by the EEA^34^, reveals only minor shifts from non-agricultural land cover classes to agriculture. In most EU Member States, these changes account for less than 0.05% of the total land area, with a maximum of approximately 0.2% observed in Portugal. In addition, ‘forest’ areas, are defined as 1 km^2^ grid cells in which the CORINE land cover classes^33^ ‘broadleaved’, ‘coniferous’ and ‘mixed’ forests cover more than 90% of a cell’s area. While these are generally considered as ‘no go’ areas, there is some flexibility to accommodate agriculture if the disaggregation model cannot allocate the agricultural areas elsewhere.

**Fig. 2:**
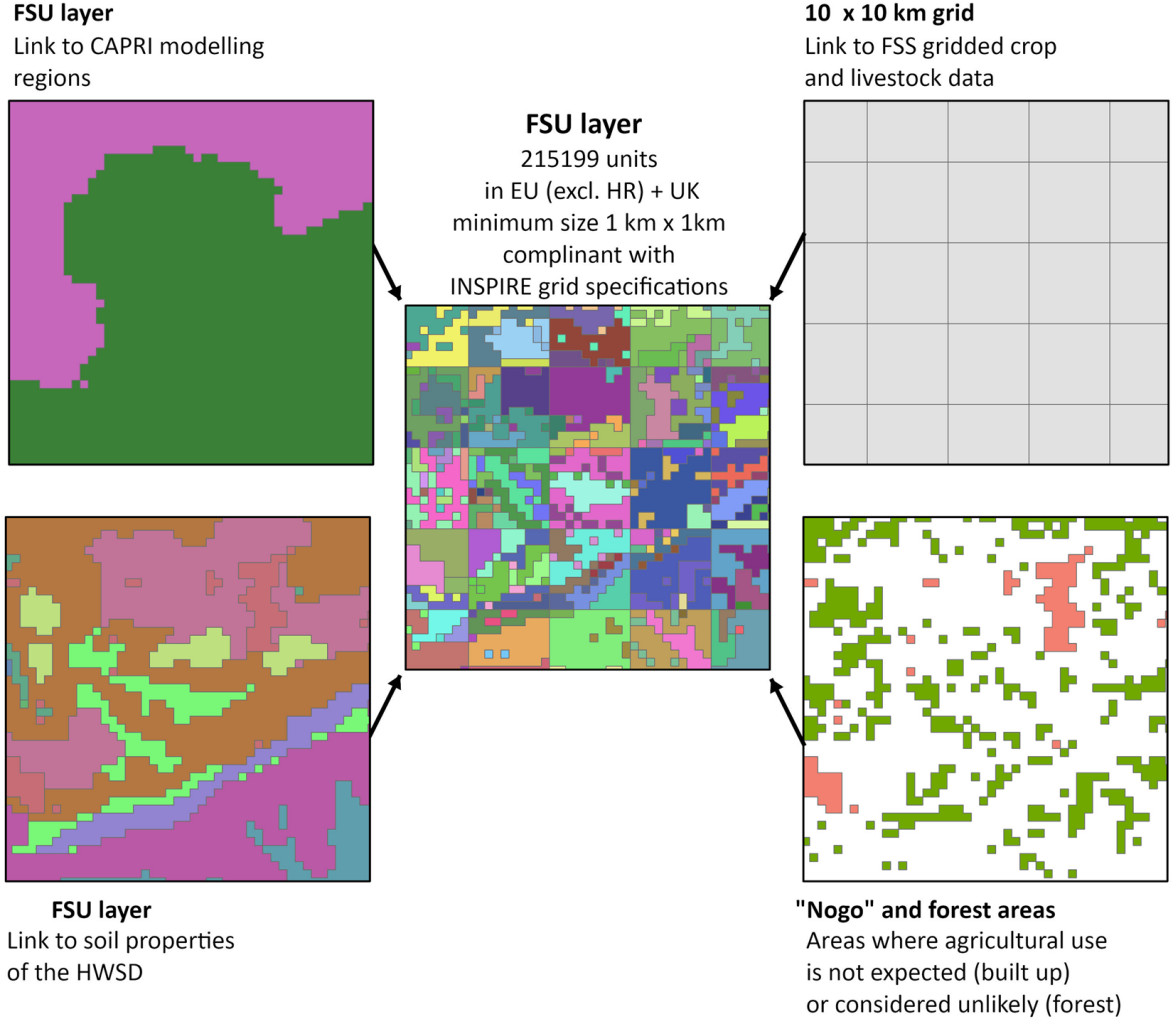
The creation of Farm Structure Units (FSUs) by intersecting spatial information for administrative units with soil data from the Harmonized World Soil Database (HWSD)^32^, the 10 km × 10 km grid and areas unlikely to host agriculture.

Figure 2 illustrates the intersection process, resulting in 215,199 FSUs with an area between 1 km^2^ and 100 km^2^ (median size 11 km^2^) in the EU (including the UK but excluding Croatia). The FSU layer is provided in the data package and is described in the ‘Data Records’ section.

### Gridded farm structure survey (FSS) data

The Farm Structure Survey (FSS)^35^, replaced by the Integrated Farm Statistics (IFS)^36^ from 2020 onwards, is an agricultural census at the level of farm holdings that takes place every 10 years across the EU and associated countries, with additional sample surveys every 3 to 4 years^37^. The information collected during the FSS of the census year 2010 covers a range of parameters including land use, livestock numbers, rural development, management and farm labour input for ~12.3 Mio farms. The data collection includes the location of the holding as latitude and longitude coordinates within a 5-minute arc. Routinely, Eurostat^38^ publishes the census data at aggregated levels such as NUTS2 or coarser, to ensure confidentiality.

For the purpose of our research project, Eurostat^28^ provided FSS 2010 farm level data on crop areas and livestock numbers aggregated at finer grid resolutions of 10 km × 10 km, 20 km × 20 km and 60 km × 60 km, as well as regional levels (NUTS3, NUTS2 and country). These experimental data sets were made available exclusively for use as a priori information in the disaggregation procedure within CAPRI.

The FSS crop and livestock data sets at all spatial aggregation levels were subject to a standard confidentiality disclosure control process applied by Eurostat before making the data available. This process involved suppressing all spatial units where the values are based on data from only 4 or fewer farms, or when more than 85% of the cell value was contributed by the 2 biggest farms. The remaining cell values were then rounded to the closest multiple of 10. This application of the disclosure rules impacts a large number of cells, masking up to 30% of the total EU value for some variables on a 10 km × 10 km grid resolution. Consequently, the regional totals did not add up. To overcome this problem, a gap filling method (for details see supplementary material) was developed to obtain a 10 km × 10 km grid data set where the crop and livestock data match the totals at the country level. For the latter, we assumed that no data had been suppressed.

Data were available for nested grids of 10 km × 10 km, 20 km × 20 km, and 60 km × 60 km, intersected with NUTS 2 regions, as well as for nested regions at country, NUTS 2 and NUTS 3 levels. The higher the resolution, the more data that were suppressed by the disclosure control process. The information from the data sets at a lower resolution was then used to gap fill the missing data in the datasets at higher resolutions. This ensured that the data at the higher resolution match the given data at the aggregated level, both spatially for nested regions and region-grid intersections, and thematically for up to six hierarchical levels (e.g., the sum of crop areas is equal to the total utilised agricultural area of the region).

As a final step in the FSS data pre-processing, the classification of crops and livestock was matched with the crop (Table 1) and livestock activities (Table 2) present in CAPRI.

**Table 1:** Matching CAPRI crop activities and FSS categories^72^.

CAPRI crop activities		FSS 2010 crop categories	
Code	Description	Code	Description
APPL	Apples pears and peaches	B_4_1^a^	Fruit and berry plantations – total
OFRU	Other fruits		
BARL	Barley	B_1_1_4	Barley
CITR	Citrus fruits	B_4_2	Citrus plantations
DWHE	Durum wheat	B_1_1_2	Durum wheat
FALL	Fallow land	B_1_12_1	Fallow land without subsidies
VSET	Set aside voluntary	B_1_12_2	Fallow land subject to payment of subsidies with no economic use
		B_3_3	Permanent grassland and meadow - not used for production, eligible for subsidies
FLOW	Flowers	B_1_8	Flowers and ornamental plants
GRAE	Gras and grazing extensive	B_3_2	Permanent grassland and meadow - rough grazing
GRAI	Gras and grazing intensive	B_3_1	Permanent grassland and meadow - pasture and meadow
MAIF	Fodder maize	B_1_9_2_1	Forage plants - other green fodder - green maize
MAIZ	Grain maize	B_1_1_6	Grain maize
NURS	Nurseries	B_4_5	Nurseries
OATS	Oats and summer cereal mixes without triticale	B_1_1_5	Oats
OCER	Other cereals including triticale	B_1_1_99	Other cereals
OCRO	Other crops crops	B_1_10	Seeds and seedlings
		B_1_11	Other arable land crops
		B_4_7	Permanent crops under glass
OFAR	Fodder other on arable land	B_1_9_1	Forage plants - temporary grass
		B_1_9_2_2	Forage plants - other green fodder - leguminous plants
		B_1_9_2_99	Forage plants - other green fodder - other than green maize and leguminous
OIND	Other industrial crops	B_1_6_12	Aromatic, medicinal and culinary plants
		B_1_6_99	Industrial plants not mentioned elsewhere
OLIV	Olive for the oil industry	B_4_3_2	Olive plantations - oil production
OOIL	Other seed production for the oil industry	B_1_6_7	Linseed (oil flax)
		B_1_6_8	Other oil seed crops
PARI	Paddy rice	B_1_1_7	Rice
POTA	Potatoes	B_1_3	Potatoes
PULS	Pulses	B_1_2	Pulses – total
RAPE	Rape	B_1_6_4	Rape and turnip
ROOF	Fodder root crops	B_1_5	Fodder roots and brassicas
RYEM	Rye and meslin	B_1_1_3	Rye
SOYA	Soya	B_1_6_6	Soya
SUGB	Sugar beet	B_1_4	Sugar beet
SUNF	Sunflower	B_1_6_5	Sunflower
SWHE	Soft wheat	B_1_1_1	Common wheat and spelt
TABO	Table olives	B_4_3_1	Olive plantations - table olives
TAGR	Table grapes	B_4_4_3	Vineyards - table grapes
		B_4_4_4	Vineyards – raisins
TEXT	Flax and hemp	B_1_6_10	Hemp
		B_1_6_11	Other textile crops
		B_1_6_2	Hops
		B_1_6_3	Cotton
		B_1_6_9	Flax
TOBA	Tobacco	B_1_6_1	Tobacco
TOMA	Tomatoes	B_1_7^b^	Fresh vegetables, melons, strawberries
OVEG	Other vegetables		
TWIN	Wine	B_4_4_1	Vineyards - quality wine
		B_4_4_2	Vineyards - other wines
UAAR	Utilized agricultural area		Utilized agricultural area
OLND^c^	Area grazed extensively outside utilized agricultural area	n.a.	n.a.

^a^The FSS class B_4_1 was split into the CAPRI classes APPL and OFRU based on the classes area shares in the CAPRI regional data.

^b^The FSS class B_1_7 was split into the CAPRI classes TOMA and OVEG based on the classes area shares in the CAPRI regional data.

^c^Class introduced for the purpose of allocating extensively grazing livestock, OLND is not a standard CAPRI activity nor an FSS category.

**Table 2:** Matching CAPRI livestock activities and FSS categories^72^, grazing livestock group and the type of digestive system.

CAPRI livestock activities		FSS 2010 livestock category		Grazing group	Digestive system
Code	Description	Code	Description		
CAFR	Female calves, raising activity	C_2_3	1 to <2 years, females	Non-dairy cattle	Ruminant
CAMR	Male calves, raising activity	C_2_2	1 to <2 years, males		
CAFF	Female calves, fattening activity	C_2_1^a^	Bovines <1 year old		
CAMF	Male calves, fattening activity				
BULL	Male adults, fattening activity, high final weight	C_2_4^b^	Bovines 2 years and older – males		
BULH	Male adults, fattening activity, low final weight				
HEIR	Heifers, raising and fattening activity (low and high final weight)	C_2_5^c^	Heifers, 2 years and older		
HEIL	Heifers, fattening activity, low final weight				
HEIH	Heifers fattening activity, high final weight				
SCOW	Suckler cow production activity	C_2_99	Bovine 2 years old and over - other cows		
DCOL	Dairy cow production activity, low yield	C_2_6^b^	Dairy cows	Dairy cows	
DCOH	Dairy cow production activity, high yield				
SHGM	Sheep and goat activity for milk production	C_3_1_1	Sheep - breeding females	Sheep and goats	
		C_3_2_1	Goats - breeding females		
SHGF	Sheep and goat activity for fattening	C_3_1_99	Sheep - others		
		C_3_2_99	Goats - others		
HENS	Laying hens production activity	C_5_2	Laying hens	Poultry	Monogastric
POUF	Poultry fattening activity	C_5_1	Poultry - broilers		
		C_5_3	Poultry - others		
SOWS	Sows for piglet production	C_4_2	Pigs - breeding sows over 50 kg	Swine	
PIGF	Pig fattening activity	C_4_1	Pigs - piglets under 20 kg		
		C_4_99	Pigs - others		

^a^The FSS class C_2_1 was split into the CAPRI classes CAFF and CAMF based on the classes area shares in the CAPRI regional data.

^b^The FSS classes C_2_4 and C_2_6 were split into the CAPRI low/high weight or yield classes (BULL, BULH and DCOL, DCOH) assuming equal shares (50:50).

^c^The FSS class C_2_5 was split into the CAPRI classes HEIR and HEIF (HEIF = HEIL + HEIH) based on the classes area shares in the CAPRI regional data. HEIF was further split into the low/high weight classes (HEIL and HEIF) assuming equal shares (50:50).

### Disaggregation of CAPRI regional data to FSUs for the base year 2017

This section describes the disaggregation process for CAPRI regional data (crop area, livestock, irrigation, crop yields as well as nitrogen inputs, losses and outputs) to FSUs for the base year 2017. In CAPRI, the ‘base year’ is defined as three-year average of the most recent years (i.e. 2016–2018 for the base year 2017) for which all necessary statistical data are fully available and verified. This base year serves as anchor point for modelling the baseline, time series and scenarios.

#### Crop area and livestock densities

**Fig. 3:**
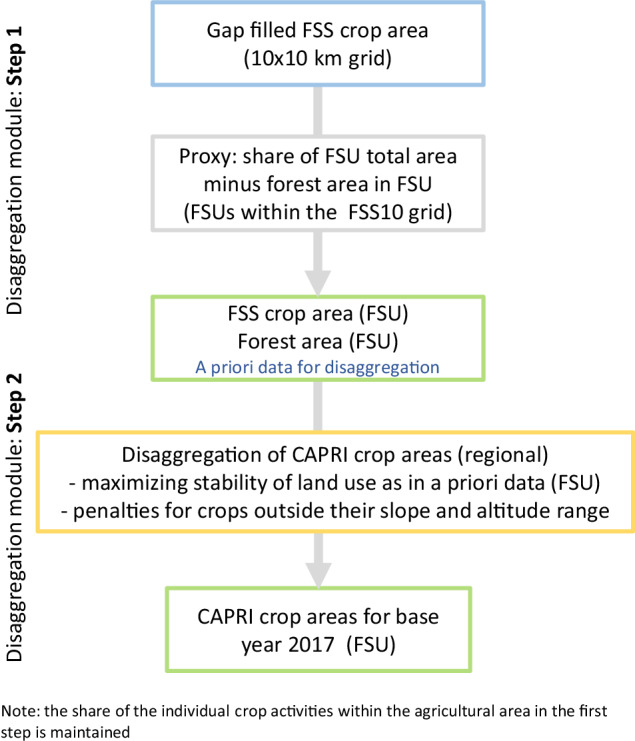
The methodological workflow to spatially disaggregate CAPRI crop class areas. Text box border colors highlight the different spatial levels (blue: 10 km × 10 km grid, yellow: CAPRI NUTS regions, green: FSU).

**Fig. 4:**
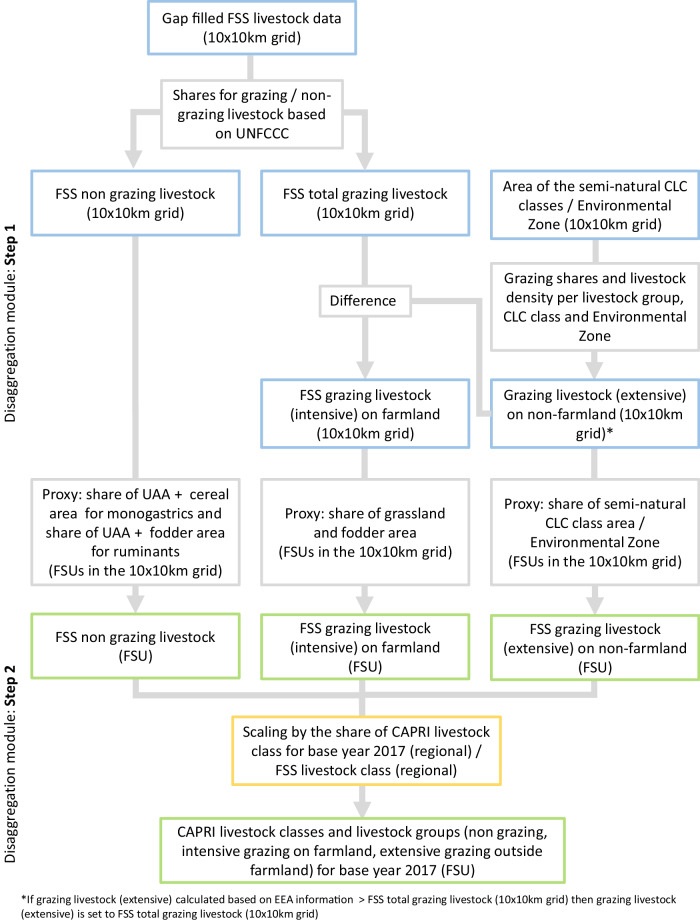
The methodological workflow to spatially disaggregate CAPRI livestock class numbers. Text box border colors highlight the different spatial levels (blue: 10 km × 10 km grid, yellow: CAPRI NUTS regions, green: FSU).

The disaggregation module implemented in CAPRI involves two general steps for both crop and livestock data. In step 1 FSS 10 km × 10 km grid data is disaggregated to the FSUs. In step 2, CAPRI regional data is disaggregated to the FSU using the results of step 1 as prior information. Figures 3, 4 illustrate the specific workflows for disaggregating crop and livestock data, respectively.

The area potentially available for agricultural land use in an FSU is defined as the area of the FSU minus the area covered by forest. The forest area within an FSU was calculated from the CORINE land cover^33^ data set. In step 1 (Fig. 3), all agricultural area from the 10 km × 10 km FSS grids were allocated proportionally to the area potentially available for cultivation in the FSU that it contained (excluding no-go FSUs). The shares of the individual crops within the agricultural area of the 10 km × 10 km FSS grids were retained.

For some 10 km × 10 km FSS grid cells, the agricultural area exceeds the area of the grid cell. This can be explained by the fact that crop areas (and livestock) in the FSS survey are allocated to the place where the farm’s administrative headquarters are registered even though the land itself may be located elsewhere, e.g., in a different grid cell or even in a different region. As it is not possible to correct these allocations, we virtually ‘inflated’ the areas of the FSUs within these FSS 10 km × 10 km grid cells, so that all forest and crop areas could be accommodated, and the sum of the agricultural area in the FSU corresponds to the agricultural area of the FSS grid cell in which it is located.

In step 2, CAPRI regional baseline data for the year 2017 is disaggregated to the FSUs by optimizing the stability of crop areas. Additionally, the disaggregation algorithm incorporates penalty terms if a significant portion of an FSU exceeds the 95th percentile of the suitable slope and altitude range for cultivating a specific crop within a country. These penalties escalate with an increasing share of the FSU area violating the suitability bounds. Consequently, the model reduces the crop area in the affected FSU and reallocates it to units with more suitable slope and altitude characteristics. The slope and altitude distribution within an FSU is calculated using high-resolution digital elevation model data from the Copernicus Land Monitoring Service (EU-DEM at a 25 m × 25 m pixel resolution)^39^.The suitability bounds for each crop type within a country were established from approximately 570,000 *in-situ* crop type observations collected during the Land Cover and Use Surveys (LUCAS)^40^, conducted between 2006 and 2018. For each crop class in every country, the 95th percentile values for both slope and altitude were derived by overlaying the LUCAS survey points with the EU-DEM data.

The procedure to distribute livestock from the 10 km FSS grid cell to the FSU distinguishes between three livestock groups: non-grazing livestock, livestock grazing on farmland (intensive grazing), and extensive grazing on non-farmland. First, we divide the total livestock numbers in the FSS 10 km grid cells into a non-grazing and grazing part. This division relies on shares of grazing animals calculated from national submissions of greenhouse gas inventories under the United Nations Framework Convention on Climate Change (UNFCCC) (Table 3B(b), Common Reporting Format)^41^, in particular the amount of nitrogen manure managed in various manure management systems and the amount of nitrogen from manure deposited in pastures, ranges and paddocks. The livestock groups for which grazing/non-grazing information is available at country level are listed in Table 2 (column ‘Grazing group’). Due to the absence of more granular data, these country-level shares are applied to all sub-national spatial units as well.

**Table 3:** Percentage of matching area (MATCHAREA) between CAPRI and GSA data for all crops, UAAR and major crop types for Austria (AT), Flanders (BEFL), Wallonia (BEWA), Denmark (DK) and France (FR).

Crops	AT	BEFL		BEWA	DK		FR
	2017	2010	2017	2017	2010	2017	2017
All Crops	76.7	74.6	77.2	74.4	81.9	76.3	74.5
UAAR	84.3	90.1	91.2	87.4	90.3	88.6	85.4
SWHE	84.1	85.2	81.7	87.1	84.1	85.2	86.8
DWHE	72.7	—	—	—	—	—	72.6
MAIF	80.7	86.9	89.7	85.3	80.9	81.1	77.9
MAIZ	84.0	82.5	89.2	67.0	55.9	32.9	80.5
BARL	83.7	84.7	79.9	77.4	87.3	87.5	82.2
OATS	77.3	—	57.0	61.1	69.6	67.9	52.1
SOYA	70.8	—	—	—	—	—	60.8
RYEM	74.4	32.3	30.5	23.6	70.6	63.9	31.8
OCER	51.5	42.1	27.2	39.9	78.9	58.6	53.3
OFAR	84.7	—	25.9	21.5	91.3	85.0	74.8
RAPE	73.1	49.1	38.1	72.1	81.5	79.9	77.7
SUNF	73.6	—	—	—	—	—	75.4
SUGB	81.9	73.8	88.3	92.5	79.6	82.8	88.8
PULS	70.8	24.7	42.5	12.4	50.8	36.4	64.7
POTA	55.5	77.9	83.4	85.7	69.2	69.7	73.8
TOMAOVEG	44.2	50.6	72.6	71.7	59.1	57.4	45.5
GRAS	77.2	76.0	86.9	73.9	75.8	76.4	70.7

In certain regions, parts of the livestock accounted for in the farm statistics may graze on semi-natural land (e.g., mountain grass-/shrub-lands or Mediterranean macchia) outside the agricultural area (non-farmland). The EEA conducted an internal study on grazing shares and livestock densities for three livestock groups (dairy cattle, non-dairy cattle, sheep and goats) in semi-natural CORINE land cover^33^ classes and different environmental zones^42^ in the EU. The data are mainly available at country level, although for Italy and Sweden, some NUTS2 level data are present. The EEA study also included the average duration of field grazing in months during the year per country. Using these data (which are provided as part of the Data Record), we adjusted the livestock density by the share of the number of months spent in the field to estimate the number of grazing livestock on non-farmland within each FSS 10 km × 10 km grid cell. First, we calculated the areas for all the CORINE land cover class and environmental zone combinations from the respective spatial layers for the FSS 10 km × 10 km grid cell. Then we multiplied the areas with the corresponding grazing shares and the adjusted densities for the three livestock groups. By subtracting the number of livestock grazing (extensive) on non-farmland from the total number of grazing livestock, we obtained the livestock grazing (intensive) on farmland for the FSS 10 km × 10 km grid cells.

In step 2 (Fig. 4), we disaggregated the livestock numbers for the three livestock groups identified in step 1 from the FSS 10 km × 10 km grid to the FSUs. This step required the agricultural crop data disaggregated to the FSU level as described above. For the non-grazing livestock, we differentiated between monogastrics and ruminants (see Table 2), taking their different dietary needs into account. Monogastrics were disaggregated to the FSUs within an FSS 10 km × 10 km grid based on their share of agricultural land plus the cereal area within the FSS 10 km × 10 km grid. While for ruminants we base the disaggregation on the share of agricultural land plus fodder crops. For intensively grazing livestock on farmland, we used the proportion of grassland plus fodder areas as a proxy. For extensively grazing livestock on non-farmland, the disaggregation was guided by the areas available for extensive grazing. These areas were calculated from the CORINE land cover and the EEA ‘Environmental zone’^42^ map for each FSU. The shares of the individual livestock categories within the livestock groups are retained. Finally, we calibrate individual livestock classes in the FSU to be consistent with the number of livestock in this class at CAPRI regional level.

#### Irrigation and crop yield

Crop yield and irrigation shares are simultaneously disaggregated from the CAPRI regional level to the FSU under the assumption that, for a given spatial unit, realized yield increases linearly from a simulated water-limited yield to a simulated potential yield as the share of irrigated area increases. Equation (1) describes the general approach to generate proxy data for disaggregating crop yields and irrigation shares:

1 $${y}_{h,c}={y}_{h,c}^{{wly}}+{f}_{h,c}^{{irri}}\cdot \left({y}_{h,c}^{{py}}-{y}_{h,c}^{{wly}}\right)\forall h,c$$

${y}_{h,c}$ Yield [kg/ha] of crop *c* in spatial unit *h* under irrigation share ${f}_{h,c}^{{irri}}$

${y}_{h,c}^{{wly}}$ Water limited yield [parameter, kg/ha] of crop *c* in spatial unit *h*

${f}_{h,c}^{{irri}}$ Irrigation share [dimensionless] of crop *c* in spatial unit *h*

${y}_{h,c}^{{py}}$ Potential yield [kg/ha] of crop *c* in spatial unit *h*

Water-limited and potential yield for 6 major crops (grain maize, spring barley, sugar beet, winter rapeseed, winter wheat, sunflower) at 0.11 degree grid resolution was available from BioMA-Wofost^43^ model simulations carried out in the frame of the PESETA project^44,45^. Data for CAPRI crops without a corresponding crop from the BioMA-Wofost model simulations are assigned the values of the crop which is assumed to be most similar. For the disaggregation we re-mapped the gridded BioMA-Wofost water-limited and potential yield data to the FSU. To ensure a complete data set, covering all BioMA-Wofost model crops and all grid cells in the region, we filled eventual data gaps with the average water limited and potential yield for all crops available in that region.

The spatial distribution of irrigated agricultural areas is derived from two main sources. The first source is the FAO irrigation map^46^, which provides irrigation shares globally on a 5 arc minute grid for the year 2005. These shares were re-mapped to the FSU. The second source is the Survey on Agricultural Production Methods (SAPM)^47,48^, which includes irrigated areas at the NUTS2 level. The SAPM data is used to calibrate the 2005 irrigations shares from the FAO irrigation map to align with official statistics from more recent years, specifically for the year 2010 in the current implementation.

The final crop yields and irrigation shares for each crop and FSU were then optimized using GAMS, to recover CAPRI total crop production and actual yield at the regional level for the base year 2017. Due to the lack of time series data on irrigation shares and BioMA-Wofost model yields, we apply the primacy of stability principle when disaggregating CAPRI times series 2010–2018.

#### Nitrogen flows (inputs, outputs and losses)

The CAPRI model calculates, at regional level (mainly NUTS2), crop-specific nitrogen inputs (mineral fertilizer, manure, biological fixation and crop residues), nitrogen outputs (harvested product and crop residues) and nitrogen losses (gaseous emissions of N_2_O, NH_3_, NO_x_ and run-off) occurring during manure management, after application of mineral fertilizer and manure to the field and resulting from manure deposited by grazing animals. Detailed information about nitrogen flows can be found in the CAPRI Online Manual^49^ and a series of dedicated publications^25,29,50–52^.

In the disaggregation, for each flow of nitrogen and crop, the sum of flows over all FSUs must recover the total flow at regional level for each crop. This applies to nitrogen inputs, outputs, and losses. The distribution of nitrogen flows at FSU level is closely tied to the previously disaggregated crop areas, yields, and livestock numbers.

Total mineral and organic fertilizer nitrogen input to a specific crop in a FSU is determined based on serveral factors: (1) the crop’s nitrogen requirements to achieve a certain yield, allowing for a certain degree of over-fertilization; (2) manure availability, taking into consideration its lower nitrogen use efficiency; and (3) ensuring that a certain fraction of the nitrogen fertilizer input is supplied by mineral fertilizer. Manure nitrogen availability in a FSU is linked to the previously disaggregated livestock numbers, excluding N losses in manure management systems (housing, storage). Manure should be applied within the same FSU whenever possible. However, to address high availability of manure in areas with high livestock densities, trade between neighbouring FSUs within the same region is permitted. This accounts for possible uncertainties in the FSS priors to allocate livestock and agricultural land and the fact, that livestock farms without land might provide the manure to neighbouring farms. Manure trade between regions, and partially also between countries, is implemented within CAPRI regional modelling and does not need to be accounted for in the disaggregation. Biological nitrogen fixation and nitrogen input from crop residues are allocated based on the area and yield of the relevant crop types in an FSU. Atmospheric deposition on cropland and grassland relies on external data from the European Monitoring and Evaluation Programme (EMEP) ‘Gridded nitrogen deposition’^53^ data set, remapped to the FSU, and is fixed. Mineralization of soil organic matter is not currently available in CAPRI regional modelling and thus is not included as a potential source of nitrogen in the disaggregation.

The allocation of nitrogen outputs via removals of the harvested product and the crop residues (primarily straw) is linked to the disaggregated yields for a specific crop.

Nitrogen losses include gaseous emissions (NH_3_, N_2_O, NO_x_) and surface run-off. Losses from housing and storage in manure management systems are allocated to the FSU based on the numbers of non-grazing livestock. Losses from mineral fertilizers and manure, applied to the field by the farmer or deposited in the field by grazing animals, are distributed proportionally to their input to the crops. We calculate the nitrogen soil surplus at the FSU level as the difference between nitrogen inputs and the sum of nitrogen outputs and losses. The definition of soil surplus provided in the disaggregated data set and the calculation of other nitrogen budgets based on the disaggregated data sets is explained in more detail in ‘Usage Notes’ section.

#### Generation of the disaggregated time series from 2000 to 2018

The disaggregated data layers for the CAPRI base year 2017 generated in the previous steps were used as priors along with regional data for 2000–2018 as constraints to produce the time series (step 3 in Fig. 1). The optimization model uses the primacy of land stability as a key operating principle. This means that if there is no indication that the land has changed (i.e., no new observations or policies restricting previous land distributions), the model tries to keep the spatial pattern similar to the patterns in the prior. This was achieved using penalties for deviations of permanent crops and forests and very high penalties when a land use type was estimated in a spatial unit when it did not exist in the prior.

## Data Records

**Fig. 5:**
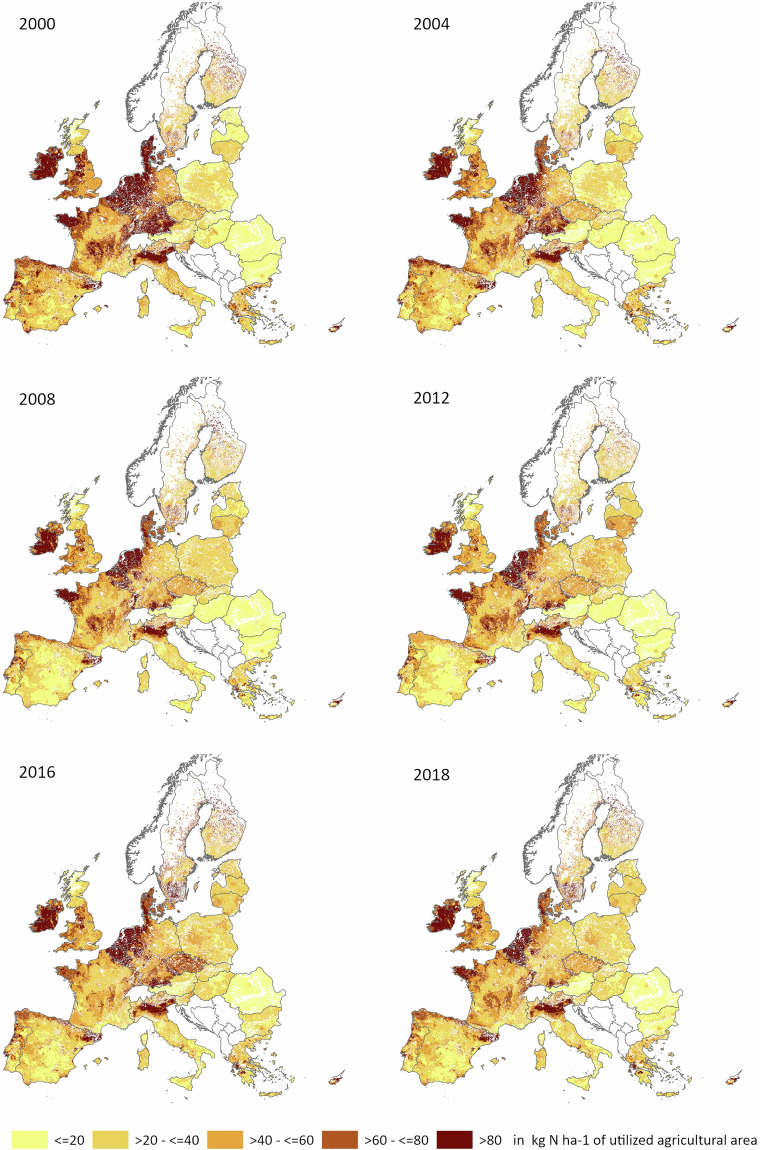
CAPRI disaggregation results for soil nitrogen surplus (leaching and denitrification, see ‘Calculating nitrogen budgets’ in section Usage Notes for details) in agricultural area for selected years between 2000 and 2018.

We provide the disaggregation results and additional background data under a Creative Commons Attribution 4.0 License at the Zenodo repository (10.5281/zenodo.13992301)^19^. The data is organized into multiple zipped data packages as given in the readme_first.txt file of our data set at the repository. Each package is supplemented by a readme file in.txt format that contains the description of the included data. The results are organized in the following.zip packages:

- fsunbuduaaolnd.zip, readme_fsunbuduaaolnd.txt: Maps of the individual nitrogen budget parameters for each year 2000 – 2018 (1 km × 1 km.tif raster files):
  - Utilized agricultural area (UAA) (ha)
  - N inputs from atmospheric deposition (kg N ha^−1^ UAA)
  - N inputs from crop residues (kg N ha^−1^ UAA)
  - N inputs from biological fixation (kg N ha^−1^ UAA)
  - N inputs from manure applied to the field by the farmer (kg N ha^−1^ UAA)
  - N inputs from manure deposited by grazing animals (kg N ha^−1^ UAA)
  - N inputs from mineral fertilizer application (kg N ha^−1^ UAA)
  - N retention (harvested product and residues removed from the field) (kg N ha^−1^ UAA)
  - N losses (gaseous emissions and run-off) in manure management systems (kg N ha^−1^ UAA)
  - N losses (gaseous emissions and run-off) following manure application and manure deposited by grazing animals (kg N ha^−1^ UAA)
  - N losses (gaseous emissions and run-off) following mineral fertilizer application (kg N ha^−1^ UAA)
  - N soil surplus (leaching and de-nitrification) (kg N ha^−1^ UAA)
  - Livestock density (livestock units ha^−1^ UAA)
  - Extensively grazed area (‘other land’) outside the CAPRI utilized agricultural area (ha^−1^)
  - N deposited by extensively grazing livestock outside CAPRI utilized agricultural area (kg N ha^−1^ ‘other land’)
- fsucroparea_ha.zip, readme_fsu_croparea_ha.txt: Maps of the areas (ha) for 35 individual crop production activities in ha (see Table 1) as separate files for each crop and each year 2000–2018 (1 km × 1 km.tif raster files).
- fsucropyield_kgperha.zip, readme_fsucropyield_kgperha.txt: Maps of the yield (in kg ha^−1^ UAA) for 37 individual crop production activities (see Table 1) as separate files for each crop and each year 2000–2018 (1 km × 1 km.tif raster files).
- fsulucategory_luperha.zip, readme_fsulucategory_luperha.txt: Maps of livestock density (livestock units ha^−1^ UAA) for 18 individual livestock production activities (see Table 2) as separate files for each livestock category and each year 2000–2018 (1 km × 1 km.tif raster files).

- fsu_lu_grazingtype.zip, readme_fsu_lu_grazingtype.txt: Maps of livestock densities for (1) total non grazing livestock (livestock units ha^−1^ UAA), (2) total livestock grazing within utilized agricultural area (livestock units ha^−1^ UAA), (3) total livestock extensively grazing outside utilized agricultural area (livestock units ha^−1^ ‘other land’) as separate files (1 km × 1 km.tif raster) for the three livestock groups and each year 2000–2018.
- fsu_delineation.zip, readme_fsu_delineation.txt: Maps of the delineation of the FSUs in vector (shapefile) and in raster (.tif) format. Although the disaggregation results for the different parameters are provided as 1 km × 1 km raster files, note that the resolution of the content is the FSU (i.e., all 1 km × 1 km raster cells belonging to the same FSU have the same value in the disaggregation results).

- EEA_extensive_grazing.zip, readme_ EEA_extensive_grazing.txt: Base information for the calculation of extensively grazing livestock outside agricultural area as.csv files. (1) Livestock densities and grazing shares for 3 livestock categories per country/region, CORINE land cover class and environmental zone, (2) the number of months per year in which extensive grazing takes place in the different countries.
  Figure 5 shows the disaggregated N soil surplus for selected years of the time series as an example.

### Notes and caveats

- In certain FSUs, the utilized agricultural area significantly exceeds the actual size of the FSU itself. This discrepancy arises from the gridded FSS data used as a prior for spatial disaggregation, where the land reported by a farmer or landowner is attributed to the location of the farm or landowner’s legal address. However, the actual land may be situated in adjoining grid cells. This situation can lead to instances where, particularly with very large farms or communal lands, certain grid cells reflect disproportionately large agricultural areas.
- For some regions in Belgium and the Netherlands, the CAPRI model estimates average nitrogen inputs from manure to agricultural areas that exceed the limits set by the Nitrates Directive^54^, which are 170 kg N ha^−1^yr^−1^, or up to 250 kg N ha^−1^ yr^−1^ if a derogation has been granted. Although the CAPRI model accounts for regional imports and exports of manure within a country, international trade is only partially implemented. The lack of comprehensive accounting for cross-border exports may result in an overestimation of actual application rates in the exporting country and an underestimation in the importing country. Unfortunately, statistical information on manure trade between 2000 and 2018 is scarce and highly uncertain, making it challenging to assess the level of error associated with not accounting for cross-border trade. Particularly in the exporting regions or countries, but not exclusively, we find extreme values for nitrogen inputs from manure in some FSUs with very high livestock density, despite the disaggregation allowing for re-allocation of manure between FSUs. Imprecisions in the gridded Farm Structure Survey (FSS) data priors contribute to an overestimation of livestock numbers in specific grid cells, which can occur when there is a mismatch between the registered location of the farm owner and the actual location where livestock is kept. In our approach, we intentionally chose not to impose a cap on nitrogen inputs from manure based on the limits set by the Nitrates Directive as suggested by other studies^14,18^. The current data limitations pose a significant challenge to re-allocating excess manure with a certain degree of confidence, particularly when a re-allocation between countries is required.
- Final results were cleaned for FSUs with very small utilized agricultural area and outliers.
- Due to the lack of gridded FSS data priors, Croatia is not included in the disaggregation.
- To reduce the impact of minor fluctuations and uncertainties in the underlying data, we recommend that users of these data sets consider calculating 3-year moving averages of the annual data sets, such as averaging the data from 2016 to 2018 to represent the year 2017. This approach can help to provide more stable and representative values, smoothing out short-term variations and yielding a more robust representation of the underlying trends.

## Technical Validation

To demonstrate the reliability of the CAPRI disaggregated database, comparisons of selected crop, livestock and nitrogen layers with data from official and other external sources were undertaken.

### Comparison of disaggregated crop areas with parcel level information

To evaluate the quality of the disaggregation of crop areas, we compared the disaggregated crop area results with high-resolution crop parcel data. This data was made publicly available by several EU countries from the Geo-Spatial Aid Application (GSA) of the CAP. The GSA is the system by which beneficiaries of funding under the CAP provide information on the crops they cultivate by agricultural parcels (or clusters of parcels with the same land use). GSA data sets were downloaded for Austria^55^ (for the year 2017), Denmark^56^ (for 2010 and 2017), Flanders^57^ (for 2010 and 2017), Wallonia^58^ (for 2017) and France^59^ (for 2017). Each country applies a unique crop classification system, which generally offers more granularity than the CAPRI classification. For the comparison, we matched the crop classes in the GSA data sets with the CAPRI classification and aggregated the crop areas to the FSU level.

To assess the alignment between GSA and CAPRI crop areas, we calculated the area where both datasets agree, the ‘true positive area (TPA) ‘, for a given crop $c$ in an FSU as:

2 $${{TPA}}_{c,{FSU}}=\min \left({A}_{{GSA},c,{FSU}},{A}_{{CAPRI},c,{FSU}}\right)$$

where ${A}_{{GSA},{c},{FSU}}$ is the area (in kha) of crop $c$ based on GSA data in the FSU and ${A}_{{CAPRI},{c},{FSU}}$ is the area (in kha) of crop $c$ based on the disaggregated CAPRI data in the FSU. We then calculated the percentage of the matching area for a single crop $c$ in a region $r$ as:

3 $${{MATCHAREA}}_{c,r}={\sum }_{{FSU}\in r}\frac{{{TPA}}_{c,{FSU}}\times 100}{{\sum }_{{FSU}\in r}{(A}_{{CAPRI},c,{FSU}})}$$

For all crops *ac* in an FSU, the true positive area (TPA) in an FSU is calculated as:

4 $${{TPA}}_{{ac},{FSU}}={\sum }_{c\in {FSU}}\min \left({A}_{{GSA},c,{FSU}},{A}_{{CAPRI},c,{FSU}}\right)$$

and the percentage of matching area for all crops ${ac}$ in a region $r$ is defined as:

5 $${{MATCHAREA}}_{{ac},r}=\sum _{{FSU}\in r}\frac{{{TPA}}_{{ac},{FSU}}\times 100}{\,\sum _{{FSU}\in {\rm{r}}}{(A}_{{CAPRI},{ac},{FSU}})}$$

where ${A}_{{CAPRI},{ac},{FSU}}$ is the area (in kha) of all crops in an FSU based on CAPRI data and corresponds to the CAPRI utilized agricultural area (UAAR).

The results for the major crop types $({{MATCHAREA}}_{c,r})$, the matching area aggregated across all single crops $({{MATCHAREA}}_{{ac},r})$ and the results when considering total UAAR as a single class, are listed in Table 3. The results for the full list of crops is provided in Tables S1-S2 in the Supplementary Material. Overall, there is a generally good correspondence between the CAPRI and the GSA data. With better alignment for single crops that occupy a larger area (such as soft wheat, fodder and/or grain maize, and barley), and poorer alignment for crops with a smaller area (e.g. vegetables) or for crop groups (e.g. pulses) for which the crop matching between CAPRI and GSA crop classes is not always unambiguous.

Figure 6 shows scatterplots comparing the CAPRI disaggregation results to the GSA data at FSU level for soft wheat for the 5 countries/regions for the year 2017 and for 2 countries additionally for the year 2010. The figures for the remaining crops are provided in the Supplementary Material (Figures S2–S8).

**Fig. 6:**
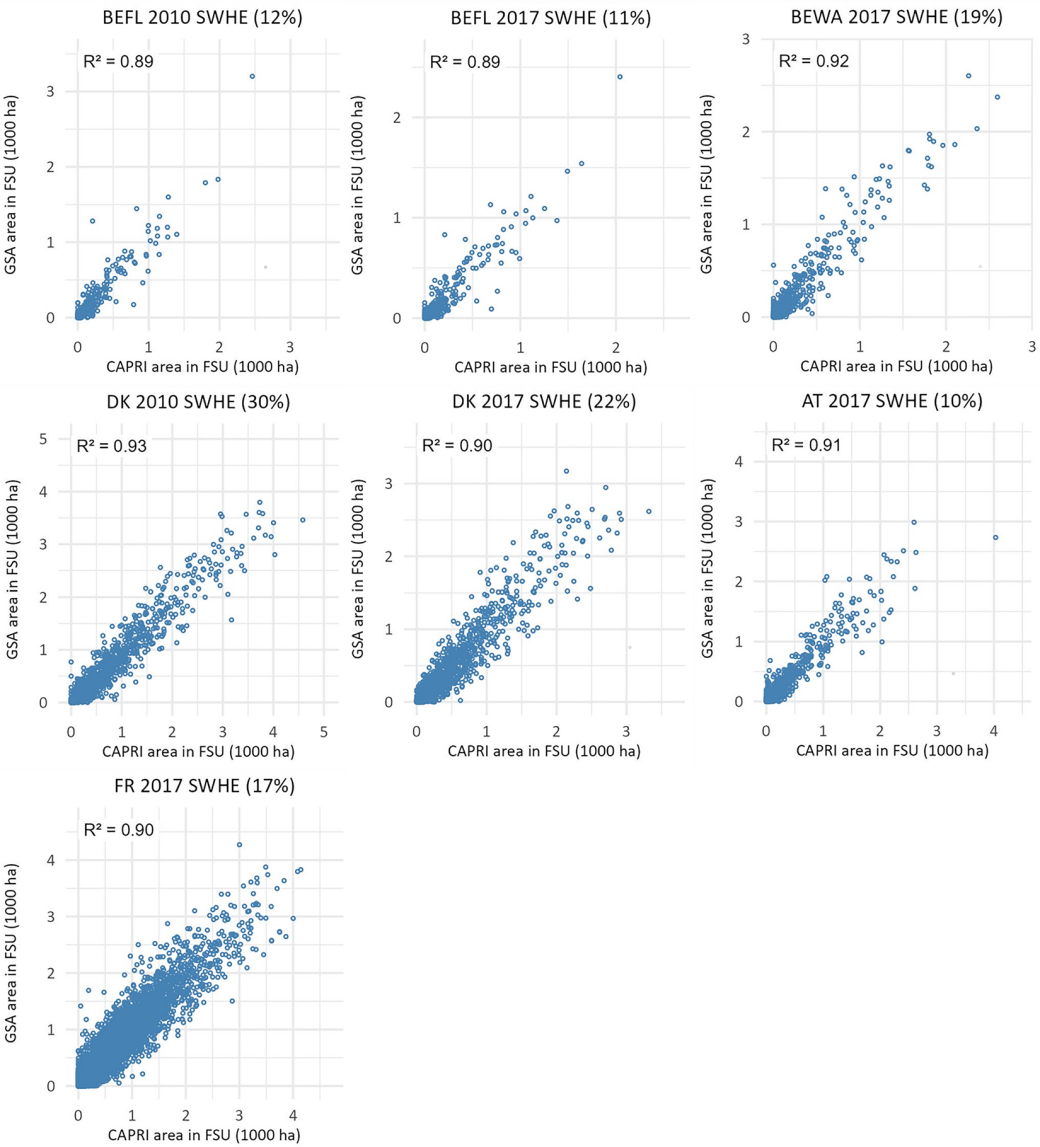
Comparison of soft wheat (SWHE) areas from the GSA data sets and from CAPRI by FSU for, Belgium Flanders BEFL (for the years 2010 and 2017), Belgium Wallonia BEWA (2017), Denmark DK (2010 and 2017), Austria AT (2017) and France FR (2017). The percentages in the scatterplot titles give the share of soft wheat in the agricultural area of the respective country/region.

For the interpretation of the results, it is important to acknowledge certain conceptual differences between the CAPRI disaggregated values and the GSA data set. First, as highlighted in the section on the crop disaggregation, the agricultural area in the FSS 10 km × 10 km grid, which is used as a proxy for the disaggregation to FSUs, should be principally interpreted as ‘Land owned by a farmer with residence in this spatial unit’. It is not ensured that all the land of this farmer is located in the same unit as well. For the same reason, we may observe FSUs in which the reported crop area exceeds the FSU area, especially if the landowner is an entity, e.g., a church with large agricultural areas dispersed over several communes. In contrast, the GSA data set reflects the actual physical area of a parcel (or a group of parcels), with a single land use reported for a given year. GSA crop areas summed at the FSU level are always smaller than or equal to the FSU area. Second, the UAAR and the area of single crops at country/regional level between the two data sets may differ. The UAAR calculated from GSA data is considerably larger for Austria (16%), Flanders (10% in 2010 and 14% in 2017) and Wallonia (10%) compared to the UAAR in CAPRI, while the opposite is the case for Denmark (−7%) and France (−2%). The latter can probably be explained by the fact that not all agricultural areas have had subsidy requests submitted for them, and therefore, these parcels are not reported in the GSA. The larger utilized agricultural area (UAAR) in Austria, Flanders and Wallonia in the GSA data set is mainly due to the areas of ‘GRASS’ (i.e., permanent grassland) and/or ‘OFAR’ (i.e., fodder on arable land, which includes temporary grassland) classes. The reason for these differences needs further investigation. Maybe some very extensively managed grasslands are not accounted for in the agricultural statistics as part of the UAAR but are nevertheless eligible for subsidies.

In addition to these conceptual differences, uncertainties may have been introduced when mapping the GSA to the CAPRI crop classification since the mapping is not always straightforward, particularly for mixed classes like fodder roots (ROOF). The GSA may list ‘beet’ as a crop type that could be a fodder root (ROOF), a sugar beet (SUGB) or a vegetable for human consumption (VEGE) in CAPRI, depending on the variety of beets and their final use. Moreover, in the case of, for example, leguminous crops such as peas, which can be harvested in the mature stage for grain (i.e., PULS in CAPRI) or at an earlier stage as green fodder (OFAR in CAPRI), the matching can be difficult if the required specification is not available in the GSA data set.

### Comparison of CAPRI disaggregated livestock with data from the FSS

**Fig. 7:**
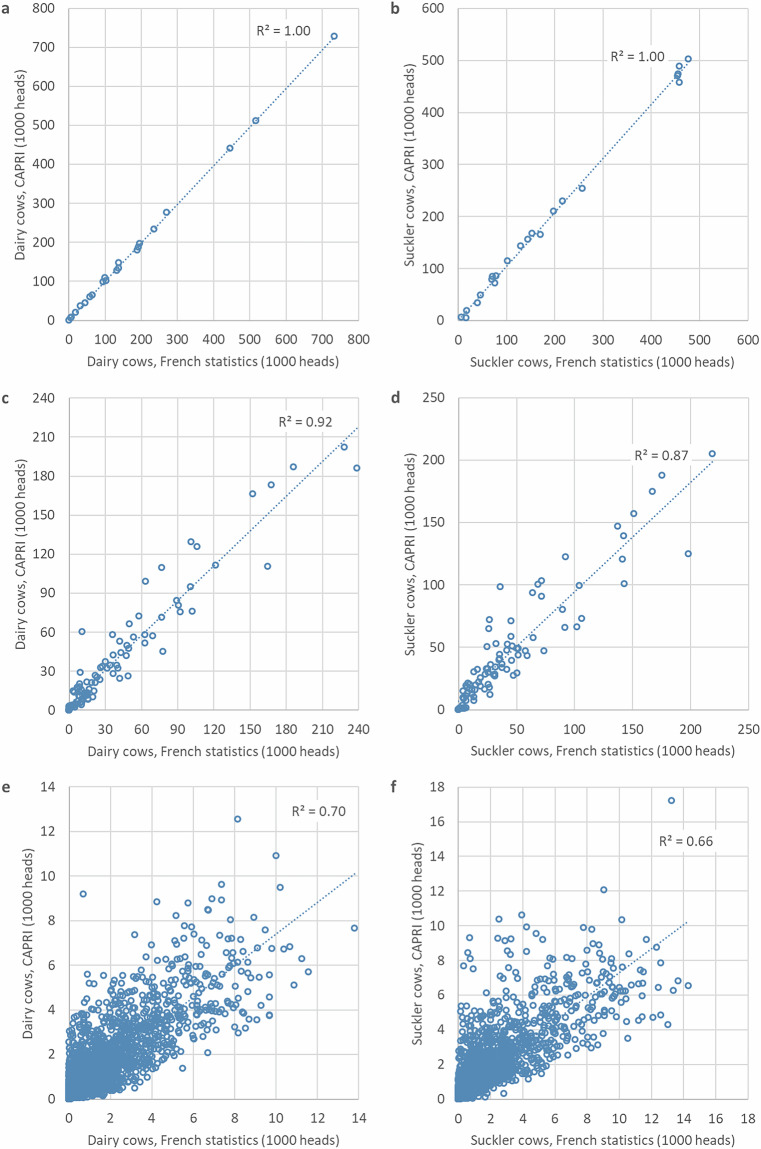
Comparison of CAPRI livestock data with FSS data for the year 2010: at regional (**a,b**), department (**c,d**) and canton (**e,f**) levels for dairy and suckler cows in France.

**Fig. 8:**
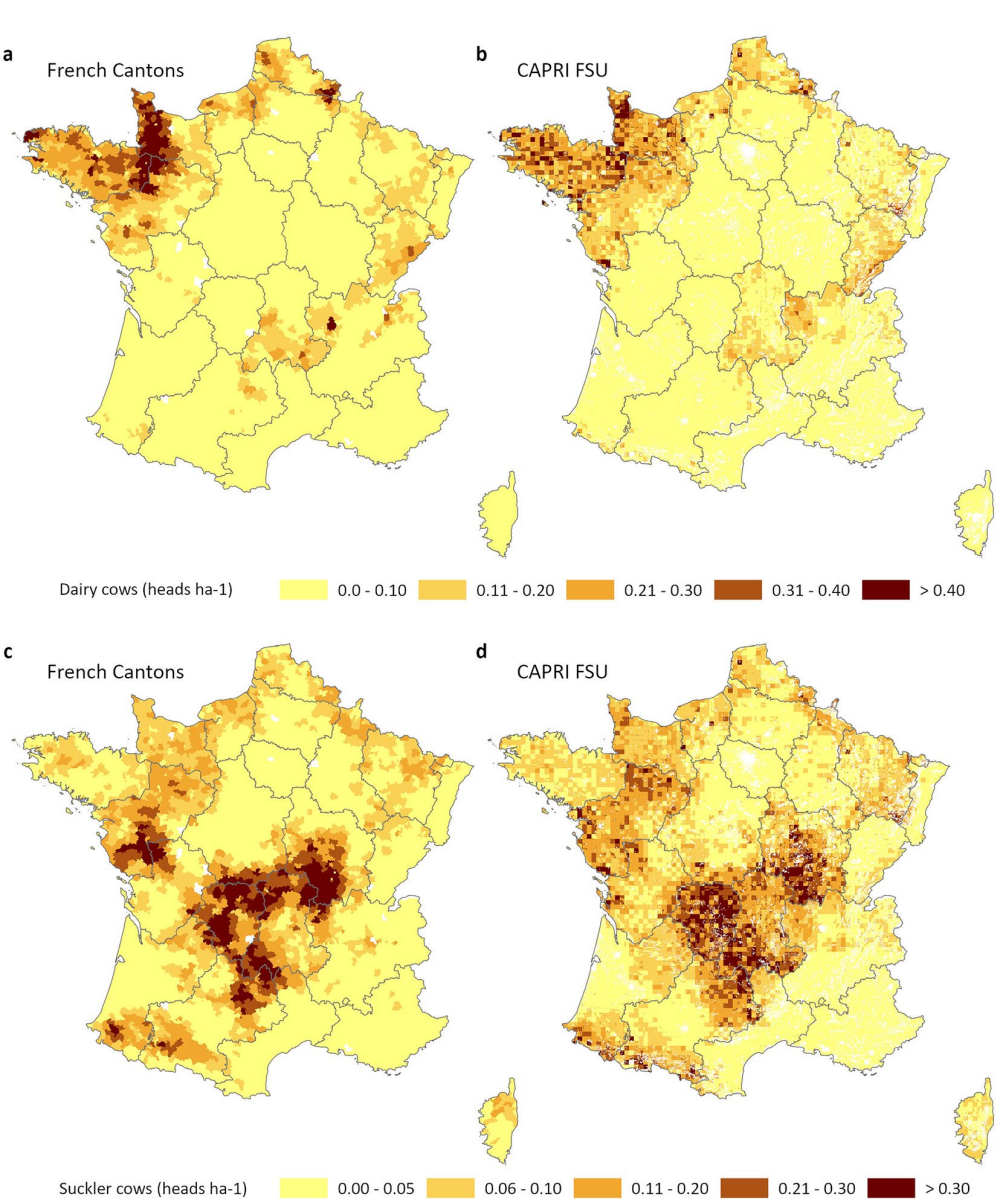
Livestock density of dairy cows (**a,b**) and suckler cows (**c,d**) based on FSS data from French cantons (**a,c**) and CAPRI data (**b,d**) at FSU level for the year 2010.

Livestock data on the number of heads of cattle are publicly available from the 2010 FSS for France at NUTS2 level from Eurostat^38^ and for NUTS3 (departments) and NUTS4 (cantons) administrative levels from the statistical service of the French ministry of Agriculture (AGRESTE)^60^. The CAPRI data were aggregated to each of these levels and directly compared with the national statistical data. Figure 7 shows scatterplots and statistical fits from these comparisons while Fig. 8 shows the spatial distribution. At country level the dairy cow numbers in CAPRI match quite well with FSS data (3.7 Mio. heads in both data sets, difference < 0.2%), and this also holds at the regional level (Fig. 7a) while for suckler cows, CAPRI livestock numbers are 4% higher (4.26 Mio. vs 4.10 Mio in FSS) with even more pronounced differences already at regional level (Fig. 7b).

At department level (Fig. 7c,d), the livestock numbers still show a good correlation, while we can see a more moderate agreement with a larger scatter at cantonal level (3673 cantons, Fig. 7e,f). Discrepanices can be driven by several aspects of the allocation scheme used in the livestock-disaggregation procedure. First, uncertainties in the factors to calculate the shares of grazing and non-grazing livestock propagate to the spatial distribution of livestock numbers. In the current approach, non-grazing ruminants are allocated as aggregated livestock group to agricultural land while keeping the original proportional shares of the constituent livestock categories (cattle, sheep, goats) within the ruminant group constant. However, aggregating all ruminants into a single class limits the ability of the method to reproduce the true spatial patterns of individual sub-categories (e.g. dairy cows vs sheep). Finally, part of the observed mismatch may originate from the way livestock are reported in the two data sets. In the Farm Survey System (FSS) livestock are recorded at the farm’s location, whereas in the CAPRI disaggregation, the allocation of livestock to the FSU level is driven by the distribution of agricultural land, grassland, and fodder-crop area.

However, we would like to highlight that total livestock numbers over all livestock categories from the FSS and CAPRI are not directly comparable. This is because CAPRI accounts for total livestock production in a year based on livestock and slaughter statistics, while the FSS looks at the livestock present at a certain reference day (which varies across countries) within the reference year^61^. For example, pig fattening requires around 4 to 5 months, and thus around 2 to 3 pigs can be raised in a row during the year in one location. CAPRI accounts for this while the FSS would not. Regarding FSS livestock reporting, Eurostat^61^ points also out that livestock numbers can fluctuate throughout the year due to seasonal effects, with e.g. sheep, goats, and pigs experiencing peak slaughter in December and January, and a decline in August and September. This may lead to inconsistencies in FSS livestock numbers among the Member States as the reference days for livestock reporting vary.

### Comparison of CAPRI nitrogen input data with other sources

**Fig. 9:**
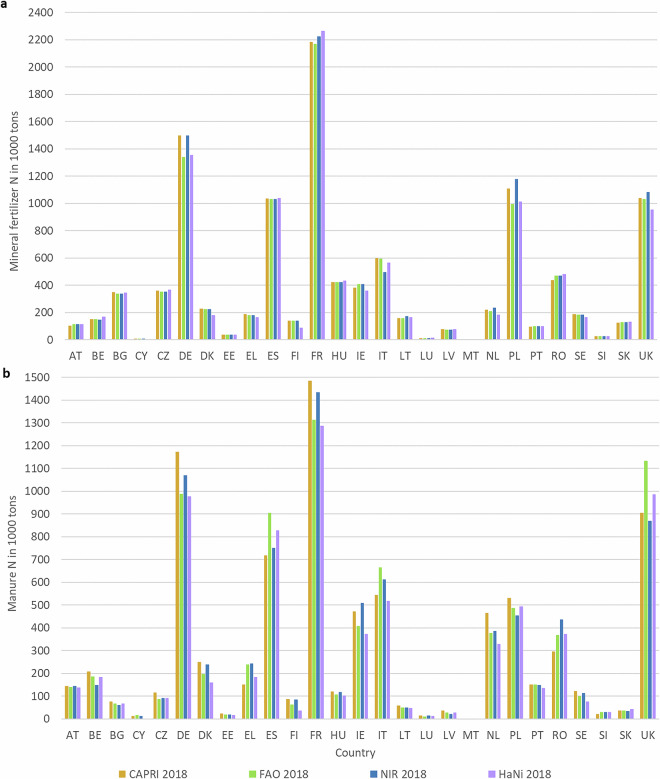
Mineral fertilizer (**a**) and manure (**b**) N input to the soil in 2018 in the EU countries and UK from CAPRI model results, FAOSTAT^62,63^, National Inventory Reports (NIR) to UNFCCC^41^ and the HaNi (History of anthropogenic Nitrogen inputs) data set of Tian *et al*.^12,13^. Manure N includes manure applied to the soil by the farmer (net of losses from housing and storage) and manure deposited by grazing animals.

The main components on the input side of the N budget in most agricultural areas in Europe are the application of N from mineral fertilizer and/or manure to the soil. To validate the N data from CAPRI, we compared the distribution of N in mineral fertilizer and manure (Fig. 9) by country for the year 2018 with data from the statistical database of FAO (FAOSTAT)^62,63^, National Inventory Reports (NIRs) to the UNFCCC^41^ and the HaNi (History of anthropogenic Nitrogen inputs) data set of Tian *et al*.^12,13^. The overall patterns show a good agreement for mineral fertilizer N between the different sources for most of the EU countries and the UK). For manure N input, which includes manure applied to the field by the farmer (net of losses during housing and storage) and manure excreted by grazing livestock, considerable differences occur among the data sets for some countries as for example Germany, Spain, France or UK.

**Fig. 10:**
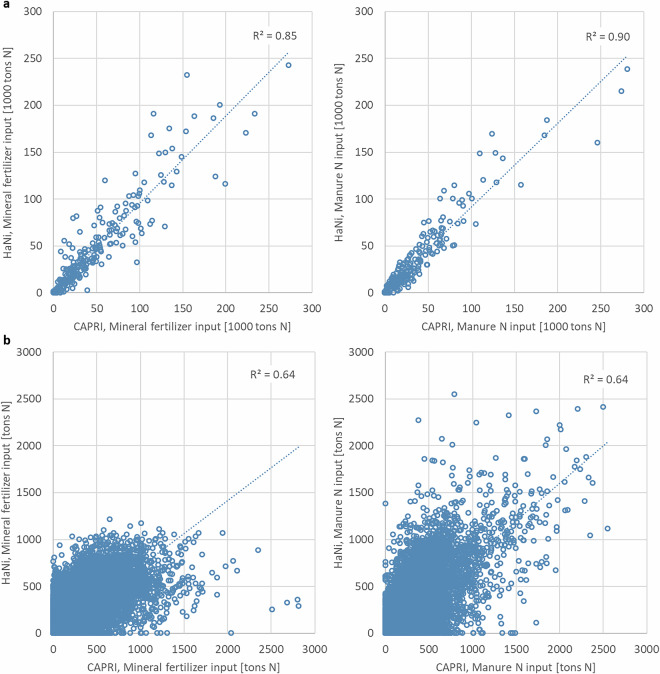
Comparison of the mineral fertilizer and manure applied to the field by the farmer and deposited by grazing animals at regional (**a**) and FSU (**b**) level in 2017 from the CAPRI model with data from the HaNi (History of anthropogenic Nitrogen inputs) data set of Tian *et al*.^12,13^.

Figure 10 provides a comparison between CAPRI N data with the HaNi data set at the regional NUTS2 (Fig. 10a) and at FSU (Fig. 10b) level for 2017. The R-squared values show good correspondence between the regional estimates, while at the finer spatial resolution of FSUs, the spatial agreement between the two data sets is not as good as for the regional comparison but the R-squared values of 0.64 still indicate acceptable agreement.

**Fig. 11:**
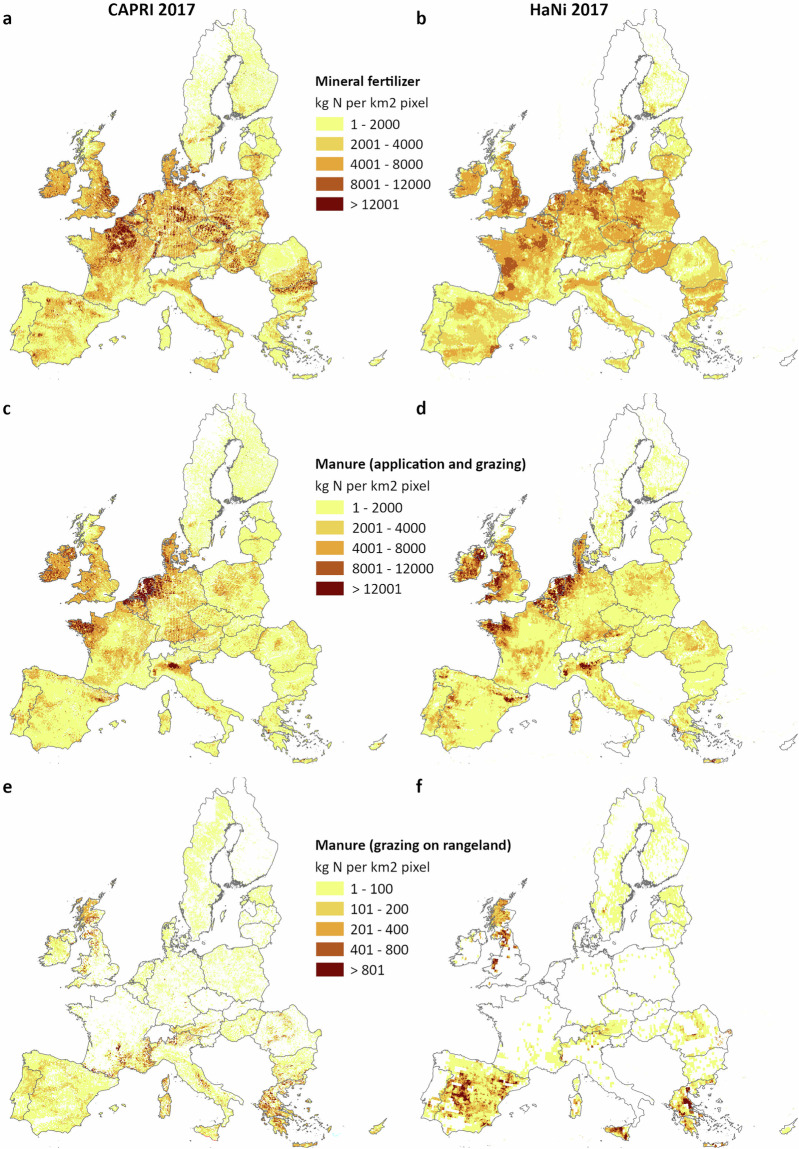
Comparison of the spatial distribution of mineral fertilizer applied to the field by the farmer, manure applied to the field by the farmer and deposited by grazing animals, and manure deposited by grazing animals on rangeland from the CAPRI model and data from the HaNi (History of anthropogenic Nitrogen inputs) data set of Tian *et al*.^12,13^ for the year 2017.

The spatial comparison between CAPRI and the HaNi data set for three N parameters in Europe is presented in Fig. 11: N from mineral fertilizer application, N from manure (application and grazing) on agricultural land and manure N deposited by extensively grazing animals on semi-natural rangelands. The overall patterns for N from mineral fertilizer are in agreement although there are clearly regional differences within countries. The comparison of N from manure (application and grazing) is overall quite similar while there are some notable differences in N input to rangelands, particularly in Spain where the values in the HaNi data set are considerably larger. These differences could be due to the estimates of outdoor grazing shares and the area of semi-natural land grazed used in the two models.

Further validation of the temporal trend of N inputs from the CAPRI model compared to other sources are provided in the Supplementary Material (Figure S9).

## Usage Notes

### Land use modelling and management

Spatially explicit data on livestock, crop yields and nitrogen budget parameters will fill a much-needed gap for researchers working in the area of land use management. For example, studies that characterize land management systems^64,65^ could use these data layers as inputs to define different intensities of management, which in turn could be used to examine impacts on biodiversity, soil organic carbon, soil erosion, etc. and their development over time.

Since these data sets align with officially reported statistics, they could be used as input data for other economic land use models to undertake ex-ante policy impact assessment. The ingestion of the data might require the re-aggregation to a coarser spatial resolution, (e.g., NUTS regions or national level) but it offers the possibility to consider the distribution of the key statistical characteristics, such as minima, maxima or percentiles, obtained from disaggregated data within these spatial levels.

### Calculating nitrogen budgets

The approach of nitrogen budgeting in agriculture is a crucial aspect of environmental management and policy because it seeks to quantify the potential for nitrogen pollution. Nitrogen, while essential for plant growth, can become a pollutant when present in excess, leading to issues such as eutrophication of water bodies, loss of biodiversity, and contamination of groundwater.

Nitrogen budgets are recognized as an environmental indicator by Organisation for Economic Co-operation and Development (OECD) and EU institutions^66^. In the framework of the European Common Agricultural Policy (CAP), a set of so-called ‘context indicators’ were selected, which provide information on the agricultural sector as well as economic and environmental trends. The ‘Gross Nitrogen Balance’ was introduced as part of context indicator C40 to address water quality in the common monitoring and evaluation framework (CMEF) of the Common Agricultural Policy (CAP) implementation 2014 to 2020 (EU Regulation 1306/2013)^67^. Under the new CAP (EU Regulations 2021/2115 and 2021/2116), which entered into force 01.01.2023, a new performance, monitoring and evaluation framework (PMEF) based on fewer, more streamlined indicators was put in place^68^. The nitrogen budget will continue to be evaluated under context indicator C39.

The disaggregated data set presented here provides all the components required to calculate a time series of land and soil nitrogen budgets at FSU or aggregated administrative level (regional, country). Table 4 (adapted from^69^) lists the input and output parameters to calculate nitrogen surplus based on the land and the soil nitrogen budget and provides the coding of the corresponding parameters of the disaggregated data set. The land nitrogen budget, which corresponds to the Gross Nitrogen Balance of OECD^70^, aims to estimate the total nutrient at risk of pollution (air, soil and water) while the soil surface nitrogen budget takes the soil surface as the boundary. Only nutrient inputs to the soil and nutrient outputs from the soil are taken into account, excluding nitrogen losses from manure management (housing, storage) occurring before the application of manure to the soil. In some variants of the soil nitrogen budget, direct emissions after the application of manure and fertilizers (nitrogen losses from volatilization) and surface run-off are excluded as well^69^. In this case, the surplus can be described as nitrogen leaching and run-off from soils and addresses the risk of ground water pollution by nitrogen more specifically. The latter is provided as parameter ‘SURSOI’ with the disaggregated data set and presented in Fig. 5.

**Table 4:** The input and output parameters from CAPRI for calculating the nitrogen surplus based on the land and the soil nitrogen budget (adapted from^69^).

Land and soil N budget parameters according to OECD (2013)	Input		Output		Surplus		CAPRI disaggregation parameter	CAPRI disaggregation parameter code
	Land	Soil	Land	Soil	Land	Soil		
Sold crop products			×	×			N export with harvest products and residues	NRET
Fodder^a^			×	×				
Crop residues			×	×				
Mineral fertilizer	×	×					N in mineral fertilizer	MINTOT ( = NMINSL + MINLOSSES)
External nitrogen sources^b^	×	×					Not considered	—
Net manure import/export and withdrawls^c^	×						Total N available from livestock excretion^g^	NMANTOT ( = NMANAP + NMANGR + MMSLOSSES + MANLOSSES)
Manure excretion	×							
Manure application^d^		×					Manure N applied to the field, net of all surface losses + Manure N deposited by grazing livestock, net of all surface losses + N losses from manure after application (NH3, N2O, NOX, runoff)	NMANAP + NMANGR + MANLOSSES
Crop residues returned to/left on the soil	×	×					N from crop residues	CRESID
Biological N fixation	×	×					N from biological fixation	BIOFIX
Atmospheric deposition	×	×					N from atmospheric deposition	ATMOSD
Soil N-stock changes^e^				×	×		Not available	—
N-gas emissions before manure application^f^					×		N losses from manure in manure management systems	MMSLOSSES
Leaching and runoff before manure application					×			
N-gas emissions and run-off from soil^f^					×	×	N losses from mineral fertilizer application (NH_3_, N_2_O, NO_X_, runoff) + N losses from manure after application (NH_3_, N_2_O, NO_X_, runoff)	MINLOSSES + MANLOSSES
Leaching and denitrification					×	×	Soil surface surplus of N (net of gaseous emissions and runoff from mineral fertilizer and manure)	SURSOI

^a^Fodder crops, cereals and other crops grown for feeding, grazed and cut grass.

^b^Sewage sludge, compost, etc.

^c^In the soil budget, manure input/export and withdrawals must be implicitly considered in the application rate. Note that for the farm and land N-budget, manure export and withdrawals are considered as negative N input with manure.

^d^Net of losses from housing and manure management systems.

^e^Soil N-stock changes are counted as a positive contribution of N surplus for farm and land budgets if the soil is depleting in nitrogen. It is counted as a positive contribution to the output of the soil N-budget if nitrogen is accumulating in the soil.

^f^NH_3_, NO_X_, N_2_O, N_2_.

^g^Net of international/regional manure imports and exports.

Based on the disaggregated data set, the nitrogen surplus based on the land budget (SURLNB) and the soil surface budget (SURSNB) can be calculated based on the following equations (see Table 4 for the parameter codes):

6 $${\rm{SURLNB}}={\rm{ATMOSD}}+{\rm{BIOFIX}}+{\rm{CRESID}}+{\rm{NMINTOT}}+{\rm{NMANTOT}}\mbox{--}{\rm{NRET}}$$

7 $${\rm{SURSNB}}={\rm{ATMOSD}}+{\rm{BIOFIX}}+{\rm{CRESID}}+{\rm{NMINTOT}}+{\rm{NMANAP}}+{\rm{NMANGR}}+{\rm{MANLOSSES}}-{\rm{NRET}}$$

**Fig. 12:**
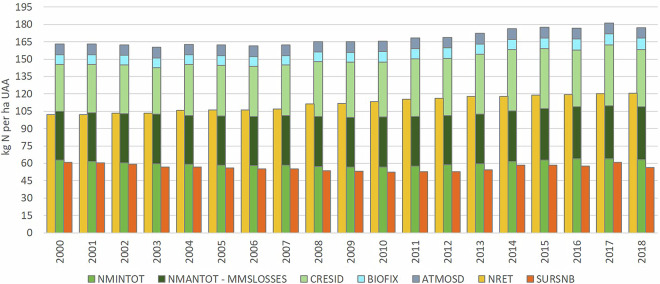
Timeseries of soil surface nitrogen budget parameters (light orange colour: outputs, green/blue colours: inputs, dark orange colour: surplus) calculated from CAPRI disaggregated data at EU level. For parameter codes see Table 4.

**Fig. 13:**
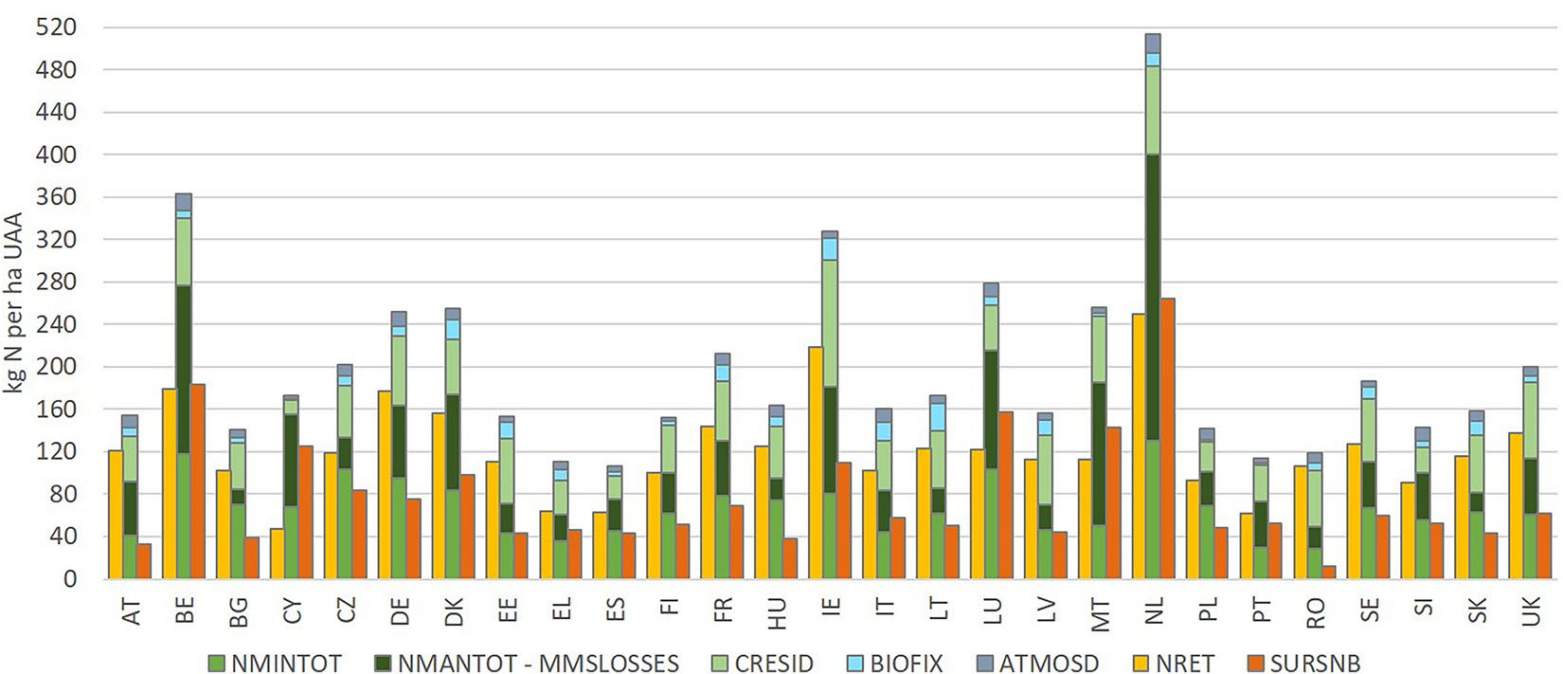
Soil surface nitrogen budget parameters at country level as average of the years 2016-2018 (light orange colour: outputs, green/blue colours: inputs, dark orange colour: surplus) calculated from CAPRI disaggregated data at EU level. For parameter codes see Table 4.

The parameters (inputs, outputs, surplus) of the soil surface nitrogen budget (SURSNB) based on CAPRI disaggregated data are illustrated in Fig. 12 (temporal development at EU + UK level) and Fig. 13 (average for the years 2016-2018 at country level).

The temporal pattern reveals almost constant nitrogen input between 2000 and 2009. From 2009 it rises steadily, reaches a peak in 2015-2017, and then declines. In our data this is driven mainly by increased mineral fertiliser use. Nitrogen surplus fluctuates but does not increase in 2017/2018 relative to 2000. This is due to a rise in yield-related nitrogen removal (NRET) exceeding the rise in nitrogen inputs, indicating an improvement in nitrogen use efficiency.

At country level (Fig. 13), the highest nitrogen inputs and associated losses occur in regions with intensive agriculture, such as the Netherlands, Belgium, and Ireland.

The larger scale nitrogen surplus (SURSNB) estimated from our dataset is consistent with values reported in recent literature. At the EU-28 level, the CAPRI-derived surplus based on the soil surface budget amounts to 52 kg N ha^−1^ UAA for 2010 (Fig. 12). By comparison, De Vries *et al*.^18^ estimate an average surplus of 53 kg N ha^−1^ UAA for the same year using the Integrator model, and Batool *et al*.^14^ report a surplus of approximately 55 kg N ha^−1^ UAA with an uncertainty range of ±2–3 kg N ha^−1^.

**Fig. 14:**
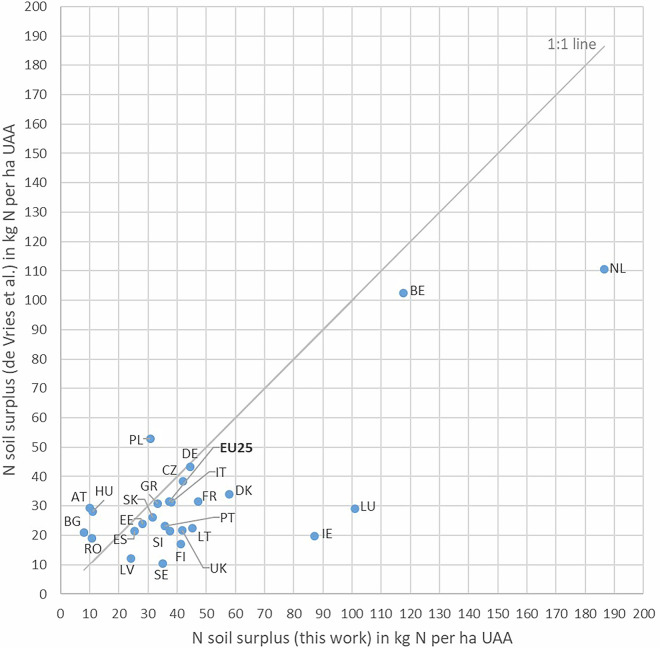
Country-level comparison of nitrogen soil surplus in agricultural area for 2010 between this study and de Vries *et al*.^71^. Nitrogen surplus is calculated as nitrogen inputs minus nitrogen removal, emissions, and surface runoff, corresponding to the parameter SURSOI (Table 4). Points are labeled with two-digit ISO country codes.

At finer spatial resolution and with a more detailed partitioning of nitrogen loss pathways, differences between our results and those reported in other studies become more pronounced. Nitrogen surplus data published at country level by de Vries *et al*.^71^ refer to the soil nitrogen surplus defined as nitrogen inputs minus nitrogen removal, emissions, and surface runoff, corresponding to the parameter SURSOI in our study (Table 4). When comparing SURSOI-based results at country scale for the year 2010 (Fig. 14), we observe systematically higher surpluses in our dataset, particularly in countries with high livestock densities such as the Netherlands, Belgium, and Ireland. At the EU-25 level (EU-27 as in 2010, excluding Cyprus and Malta), CAPRI-based disaggregation yields an average surplus of 37.3 kg N ha^−1^ UAA, which is 19% higher than the 31.4 kg N ha^−1^ UAA reported by de Vries *et al*.^71^ based on Integrator model results at the resolution of their basic spatial units (NCUs; ~40,000 units across their EU model domain). As the EU-28 average nitrogen surplus based on SURSNB is nearly identical between de Vries *et al*.^71^ and our study, the divergence in SURSOI-based results likely reflects methodological differences in the assumed partitioning of nitrogen losses among gaseous emissions, surface runoff, and below-ground pathways (i.e., above and below the soil surface).

A conclusive assessment of the spatial differences and their underlying drivers would require a detailed comparison of input data, parameterization of loss and removal terms, and spatial allocation procedures at the most disaggregated spatial level. Such an analysis remains challenging because, although the underlying estimates are derived from high-resolution assessments, the published data are predominantly available only in aggregated form, either at administrative level^14,18,71^ or as totals across land-use categories^14^.

## Supplementary information

Supplementary Material

## Data Availability

The disaggregation results and additional background data are available under a Creative Commons Attribution 4.0 License at the Zenodo repository (10.5281/zenodo.13992301)^19^.

## Code availability

The CAPRI model including the disaggregation module is available for public download at https://www.capri-model.org/doku.php?id=capri:install. For the disaggregation results presented here, we used the CAPRI trunk version (revision 10667, downloaded on 10.03.2023). This version is available on request as it requires an account on a subversion (SVN) server hosted at Bonn University. Access contact: https://www.capri-model.org/dokuwiki/doku.php?id=capri:contact. CAPRI is based wherever possible on free software. An important exception is the GAMS (General Algebraic Modeling System) modelling language. The methodology of CAPRI requires performing and stable software able to solve large-scale non-linear optimization problems, and GAMS offers interfaces to these so-called solvers. Additionally, GAMS is a language designed specifically to support economic modelling. The CAPRI Online Manual (https://www.capri-model.org/dokuwiki_help/doku.php) guides through the conceptual and technical specifications of the CAPRI model.
