# Supplementary material for: EU Wide Disaggregated CAPRI Model Data: Crops, Livestock, Nitrogen In- and Outputs (Timeseries 2000-2018)
Source: Sci Data. 2026 Mar 5;13:1085. doi: 10.1038/s41597-026-06919-8 (PMC13396626; doi:10.1038/s41597-026-06919-8)
Supplement: Supplementary file 1 — Supplementary Material [file 41597_2026_6919_MOESM1_ESM.docx]

**Supplementary Material to Data Set: EU Wide Disaggregated CAPRI Model Data: Crops, Livestock and N Inputs and Outputs (Timeseries 2000-2018)**

Renate Koeble^1^, Adrian Leip^2^, Markus Kempen^3^, Jan-Erik Petersen^4^, Oscar Gomez^5^, Debbora Leip^6,7^, Rui Catarino^9^, Maria Bielza^8^, Franz Weiss^9^, Xavier Rotllan-Puig^10^, Maria Luisa Paracchini^9^, Linda See^11^, and Marijn van der Velde^9^

**Affiliations**

1 Arhs Developments S.A., 4370 Belvaux, Luxembourg

2 European Commission, DG Research and Innovation, 1049 Brussels, Belgium

3 EuroCARE, 53123 Bonn, Germany

4 European Environment Agency, 1050 Copenhagen, Denmark

5 European Commission, EuroStat, 2721 Kirchberg, Luxembourg

6 Potsdam Institute for Climate Impact Research - Member of the Leibniz Association, 14412 Potsdam, Germany

7 Department of Agricultural Economics, Humboldt-Universität zu Berlin, 10115 Berlin, Germany

8 Independent researcher (Seidor), 21027 Ispra (VA), Italy

9 European Commission, DG Joint Research Centre, 21027 Ispra (VA), Italy

10 ASTER-Projects, 08694 Guardiola de Berguedà, Spain

11 International Institute for Applied Systems Analysis, 2361 Laxenburg, Austria

*Corresponding author(s): Renate Koeble (renate.koeble@ext.ec.europa.eu and Marijn van der Velde marijn.van-der-velde@ec.europa.eu)

### The CAPRI model

The CAPRI (Common Agricultural Policy Regionalised Impact) partial-equilibrium model is a tool for ex-ante impact assessment of agricultural and international trade policies^1^ including regional analyses of Common Market Organisations (e.g., sugar, dairy), the trade of EU agricultural goods internationally, (e.g., WTO proposals), the impact of environmental policies (e.g., greening, climate action and water) and the effect of different subsidy schemes in Europe (e.g. partial decoupling of agricultural subsidies)^2^. Other studies using the CAPRI model have included the quantification of nitrogen budgets for agriculture in the EU at the farm, landscape and soil levels including the main drivers of nitrogen surplus and nitrogen use efficiency^3^, the calculation of net GHG emissions from the EU livestock sector^4^ and more recently, the impact of the EU’s Farm to Fork Strategy to reduce fertilizer and pesticide use, reduce biodiversity losses and increase organic farming^5^.

The focus of the model is the European Union (EU), but it includes international trade flows with countries (and regional aggregations) outside of the EU via a market module that incorporates tariff quotas and subsidized exports. The supply module consists of independent aggregate non-linear programming models representing activities of all farmers at regional (mainly NUTS2) or farm type level captured by the Economic Accounts for Agriculture (EAA)^1^.

In the past, the CAPRI model has been used for various agricultural, environmental and climate policy impact and evaluation exercises in the European Commission (EC) by DG-AGRI, DG-ENV, DG-CLIMA, Eurostat and the JRC^2^. More specifically, it is used to analyse different economic and environmental policy scenarios regarding reforms to the CAP. For example, the CAPRI model has been used to examine the impact of the greening of the CAP on the area of crops grown, crop prices and farm incomes^6^. In the post-2013 CAP reform, three greening activities were introduced: preservation of permanent grasslands, crop diversification and implementation of ecological focus areas. The model results showed that the CAP reform had minimal impact on agriculture in the EU. The main effect would be a decrease in arable land and production, resulting in price increases in EU markets that would be passed onto consumers in the form of higher food prices. However, the price increases compensated for lower production, resulting in slightly higher farm incomes.

Over the last decade, the CAPRI model has also been expanded to explicitly include irrigation in the supply module, allowing differentiation of crop shares into irrigated and rainfed and taking the water needs of livestock into account^7^. The CAPRI model was then used to analyse the impact of increased prices for irrigation water in 2030, which led to a decrease in irrigated areas in southern and eastern Europe, while the overall impact of climate change was negative, leading to an increase in producer prices at both the global and EU levels.

### Gap filling of gridded Farm Structure Survey (FSS) data

The gap filling method, aims to estimate the magnitude of the supressed data for the smallest available units (i.e. NUTS 3 and a 10 x 10 km grid intersected with NUTS 2), and additionally to estimate data for the intersection of the 10 x 10 km grid intersected with NUTS 3 regions. It uses the GAMS (General Algebraic Modelling System) software.

The method assumes that geographically no data are suppressed at NUTS 0 level. Thematically the total utilised agricultural area and the total number of animals are assumed to be complete at NUTS 0 and NUTS 2 level. In these cases, all zeros are taken as true zeros. Even for these data, inconsistencies can happen due to rounding. Therefore, the method starts by scaling these values to ensure consistency with the data at a higher aggregation level.

In the next step, gaps at the first geographic subdivision (NUTS 2) are filled so that consistency is ensured. To this end, the GAMS program solves an optimisation model for each NUTS 0 region *reg_E_* and crop and livestock category *h_E_*, going from high level categories towards lower level categories, with the following constraints:

$$\forall reg_{e} \in reg_{E}:\sum_{h_{e}\in h_{E}} data(reg_{e}, h_{e}) = data(reg_{e}, h_{E})$$

*for all sub-regions (reg_e_) belonging to a region (reg_E_), the sum of the data values over all sub-categories (h_e_) in the region (reg_e_) must equal the corresponding value of the category (h_E_) in the sub-region (reg_e_)*

$$\forall h_{e} \in h_{E}:\sum_{reg_{e}\in reg_{E}} data(reg_{e}, h_{e}) = data(reg_{E}, h_{e})$$

*for all sub-categories (h_e_) belonging to category (h_E_), the sum of the data values over all sub-regions (reg_e_) for the sub-categories (h_e_) must equal the corresponding data value in the whole region (reg_E_)*

However, as there are two distinct spatial subdivisions of NUTS 2 regions (i.e., NUTS 3 and the intersection of NUTS 2 with grid cells of 60 x 60, 20 x 20, and 10 x 10 km), filling gaps in further geographical subdivisions in an top-down process can lead to inconsistencies between gridded data and data on the NUTS 3 level: Given a 10 x 10 km grid cell intersected with NUTS 2 that fully lies within a specific NUTS 3 region, assigning a value based on constraints given by the NUTS 2 region might result in a larger value than reported for the respective NUTS 3 region. Therefore, the spatial layer of 10 x 10 km intersected with NUTS 3 was inserted as a connecting link. This circular dependency between the nested regions (see Figure S1, right side) allows us to fully leverage the data provided to derive consistent estimates of the supressed data, by solving the model with the above defined constraints for all regions involved in parallel rather than top-down.

The optimization model also includes a penalty term for deviating both from the given values and from the calculated start values. The penalty term is minimised. To obtain the start values, first the difference between the *h_E_* and the sum of the respective *h_e_* is calculated for each sub-region *reg_e_*. This difference is then distributed to those sub-categories *h_e_* that did not have any assigned value, using the share in the region *reg_E_* of the sub-category *h_e_* to the sum of all sub-categories *h_e_* that are missing in the sub-region. This is done in a top-down approach, using the spatial dependency structure shown in Figure S1, left side. Deviating from a given value is penalised stronger than deviating from a calculated start value, but has to be allowed to prevent infeasibility. The GAMS code for the gap filling model is available at <https://github.com/deleip/FSSgapfilling>


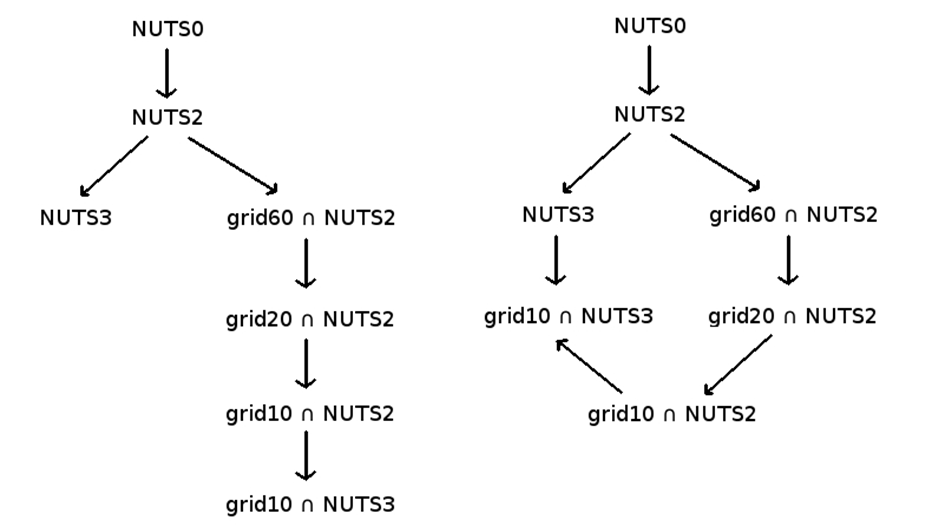


Figure S1: Structure of regions used for calculating start values (left) and used for solving the model (right)

### Technical Validation

This section contains some additional validation exercises that were undertaken to demonstrate the reliability of the CAPRI disaggregated database.

**Comparison of disaggregated crop areas with parcel level information**

Figure S2 to Figure S8 provide comparisons between parcel level crop data from the Geospatial Application (GSA)^8,9,10,11,12^ with data from CAPRI at the level of Farm Structure Units (FSUs) for those crops with the largest shares in each country/region. The share of the crop in total agricultural area of the specific country or region is given in round brackets in the title of the individual scatterplots.


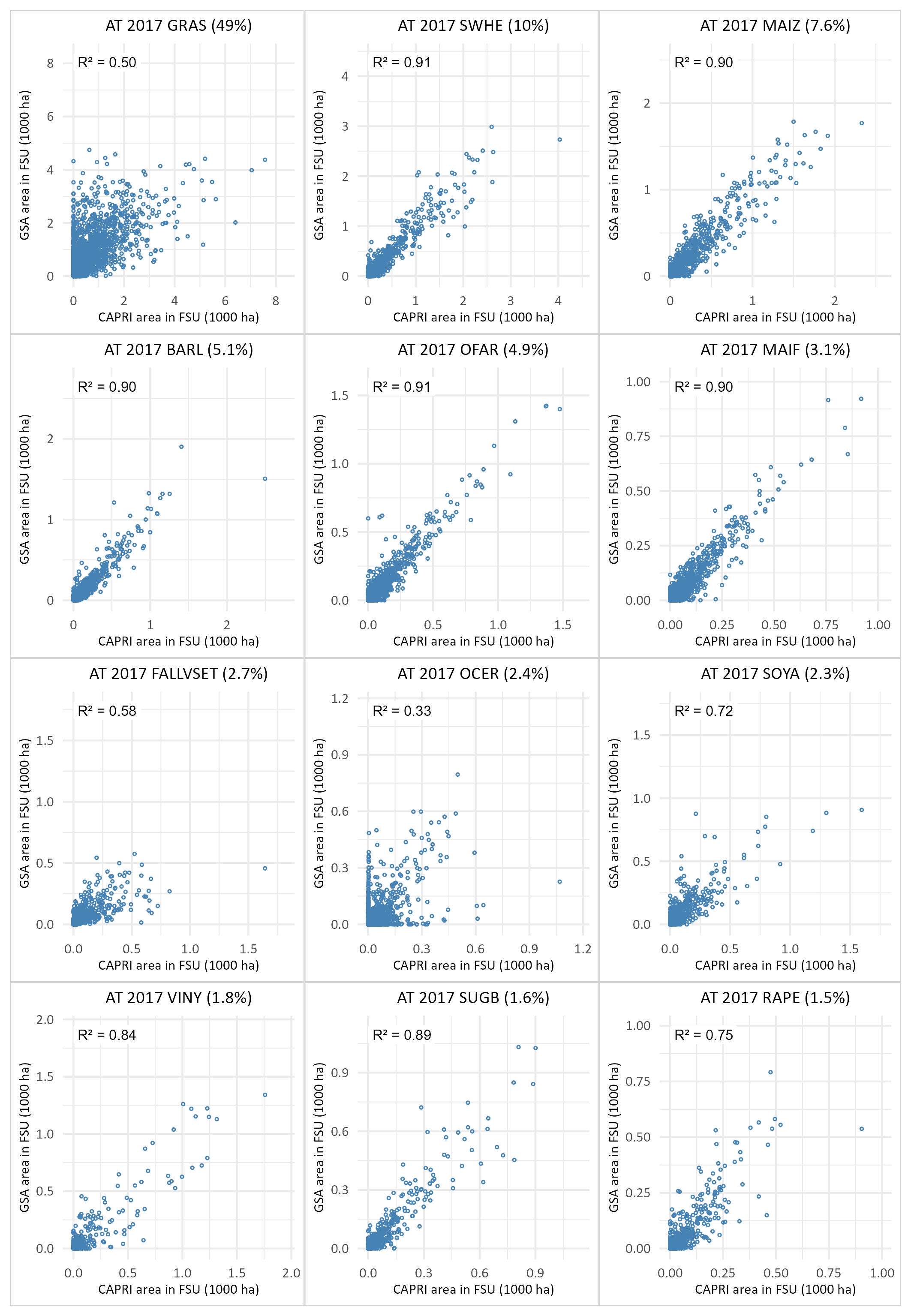


Figure S2: Comparison of the crops with the largest shares in Austria from the GSA with the same crops from CAPRI by FSU for the year 2017. The meanings of the crop codes can be found in Table 1 of the main paper.


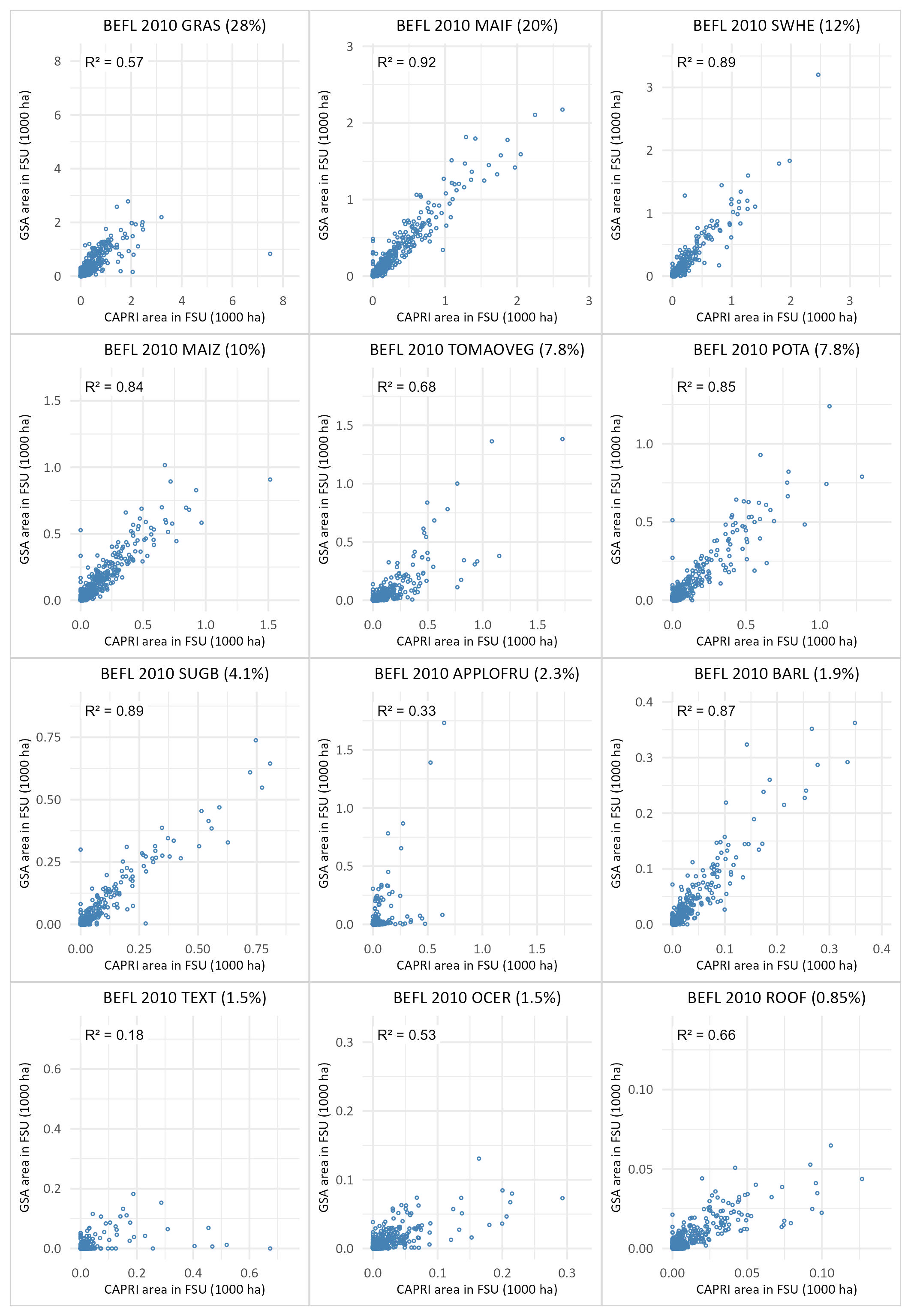


Figure S3: Comparison of the crops with the largest shares in Flanders, Belgium from the GSA with the same crops from CAPRI by FSU for the year 2010. The meanings of the crop codes can be found in Table 1 of the main paper.


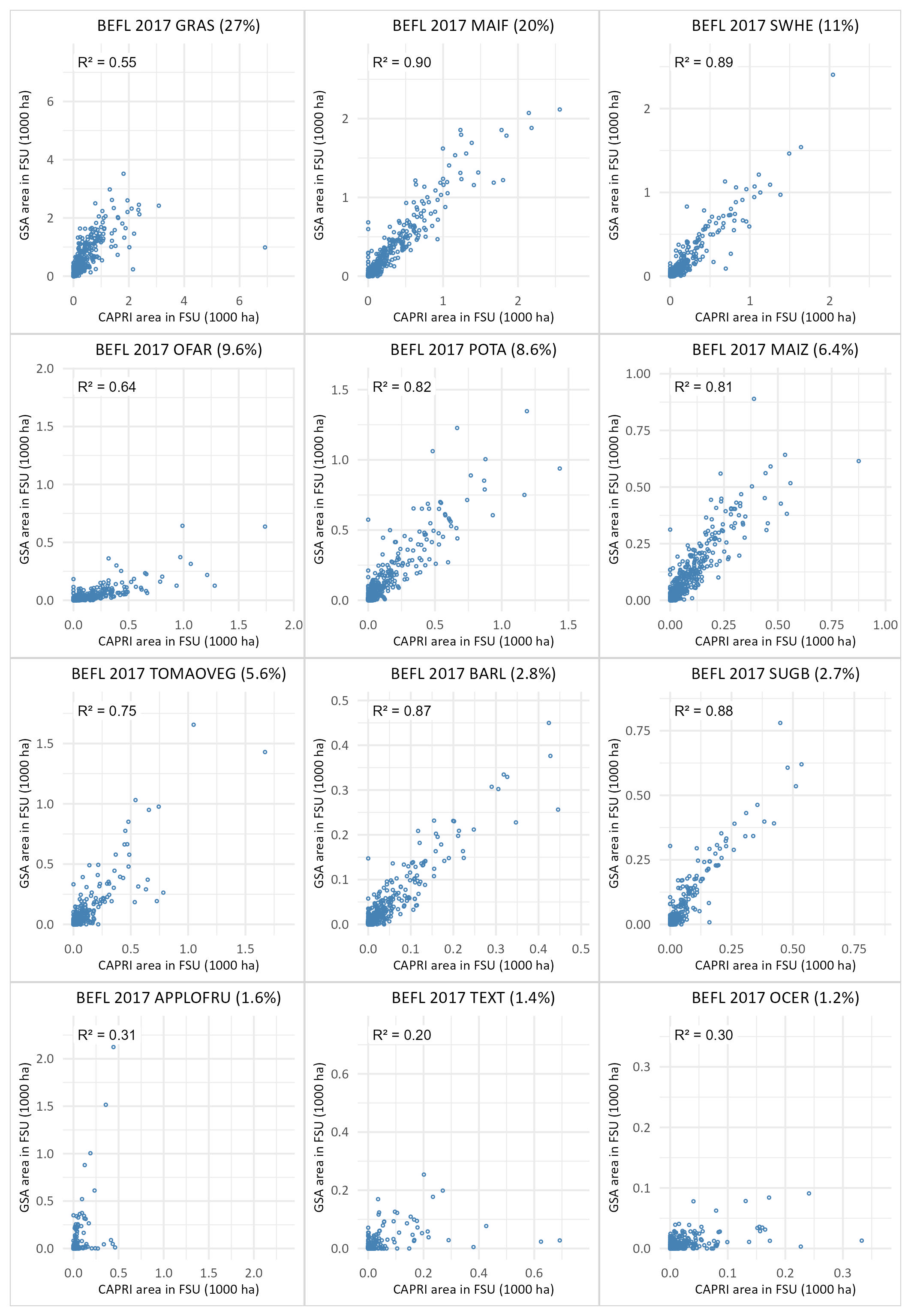


Figure S4: Comparison of the crops with the largest shares in Flanders, Belgium from the GSA with the same crops from CAPRI by FSU for the year 2017. The meanings of the crop codes can be found in Table 1 of the main paper.


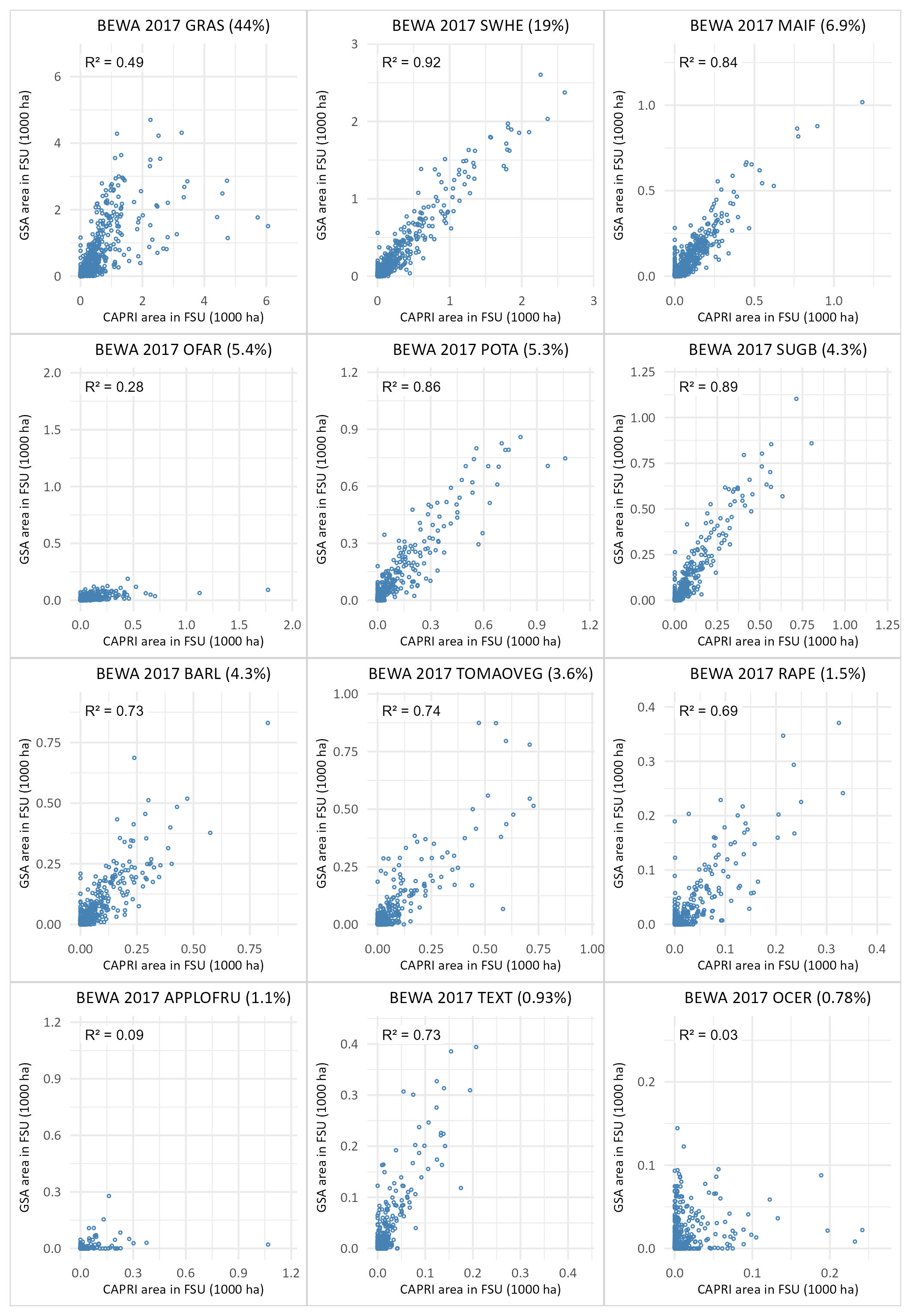


Figure S5: Comparison of the crops with the largest shares in Wallonia, Belgium from the GSA with the same crops from CAPRI by FSU for the year 2017. The meanings of the crop codes can be found in Table 1 of the main paper.


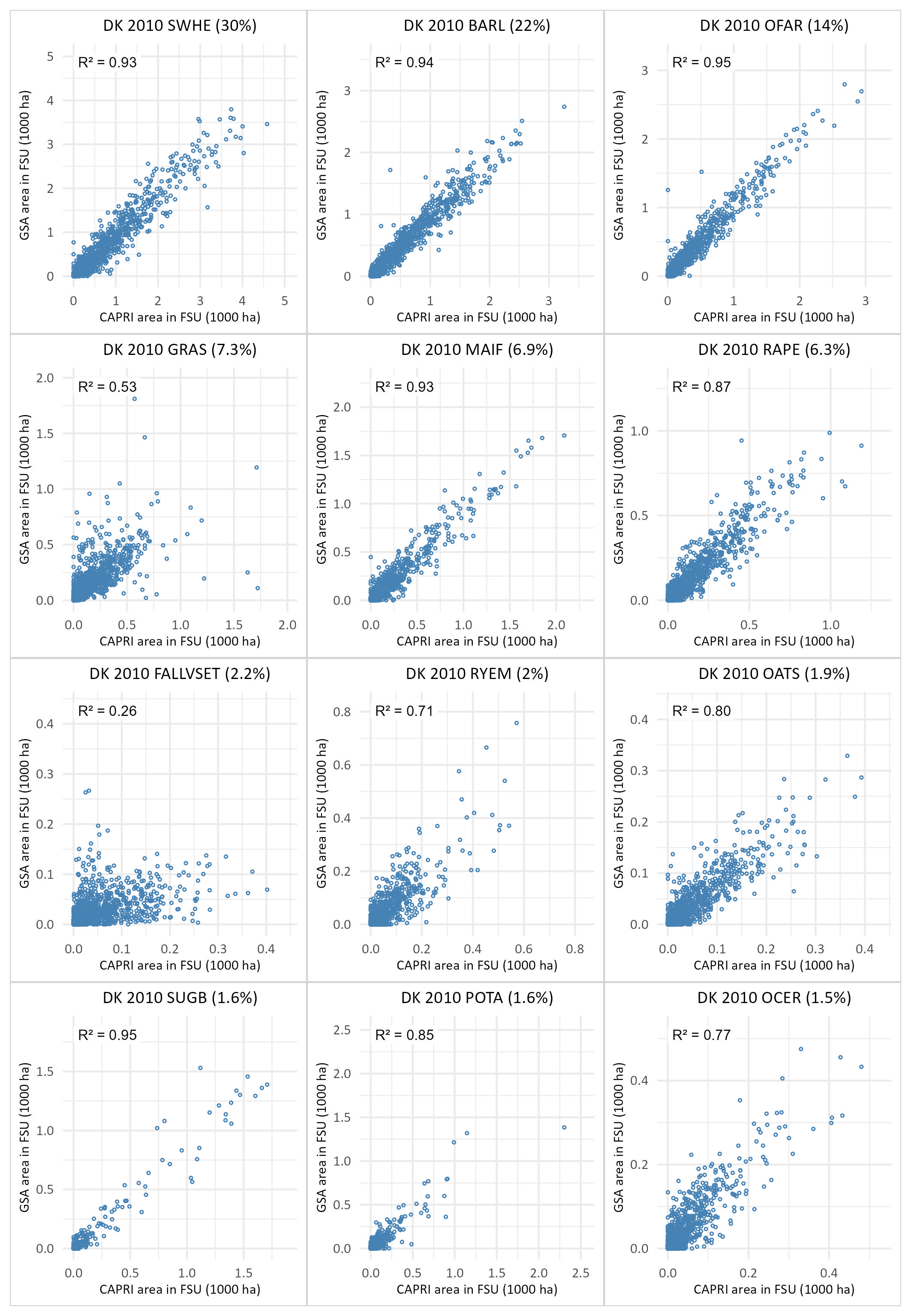


Figure S6: Comparison of the crops with the largest shares in Denmark from the GSA with the same crops from CAPRI by FSU for the year 2010. The meanings of the crop codes can be found in Table 1 of the main paper.


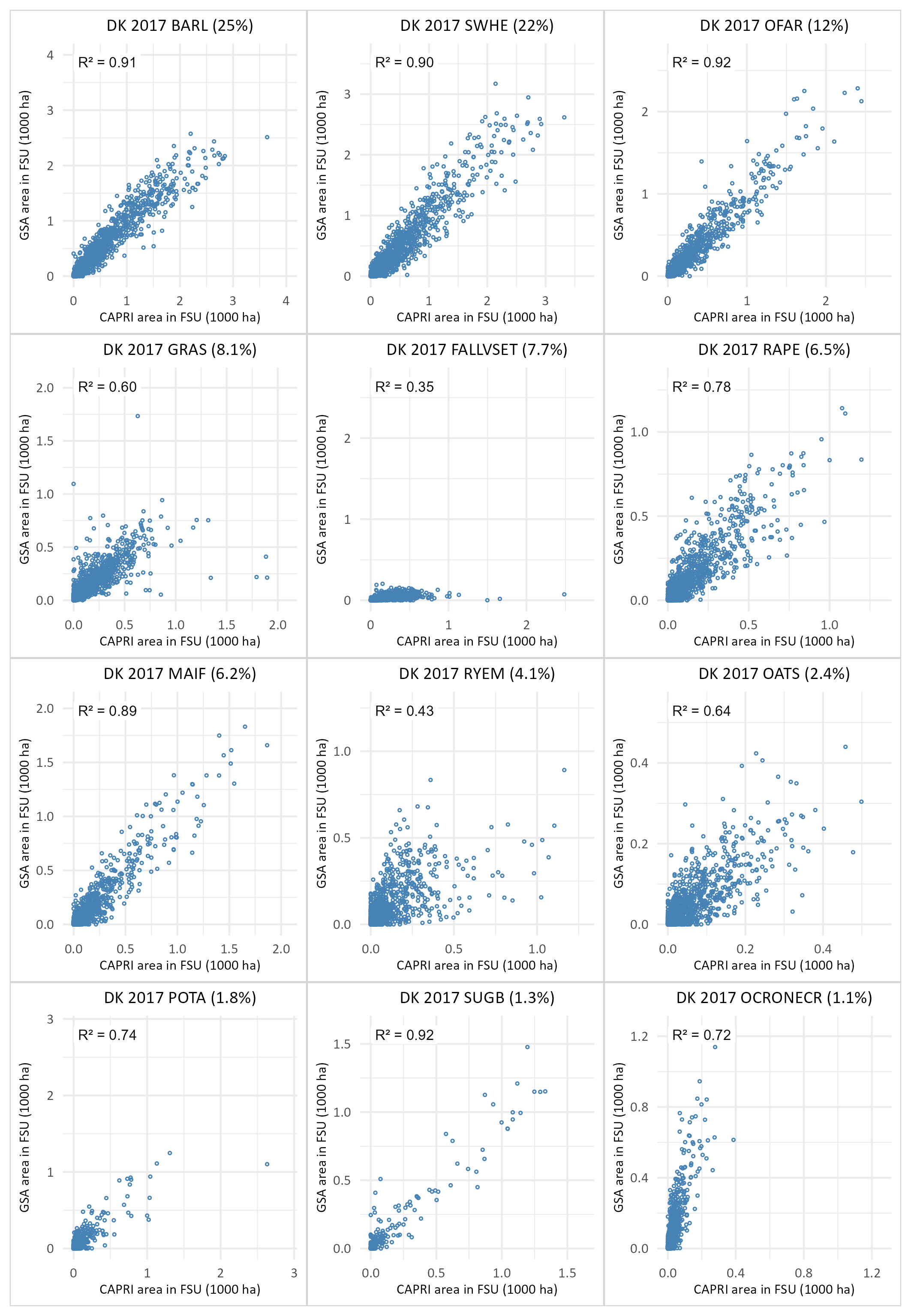


Figure S7: Comparison of the crops with the largest shares in Denmark from the GSA with the same crops from CAPRI by FSU for the year 2017. The meanings of the crop codes can be found in Table 1 of the main paper.


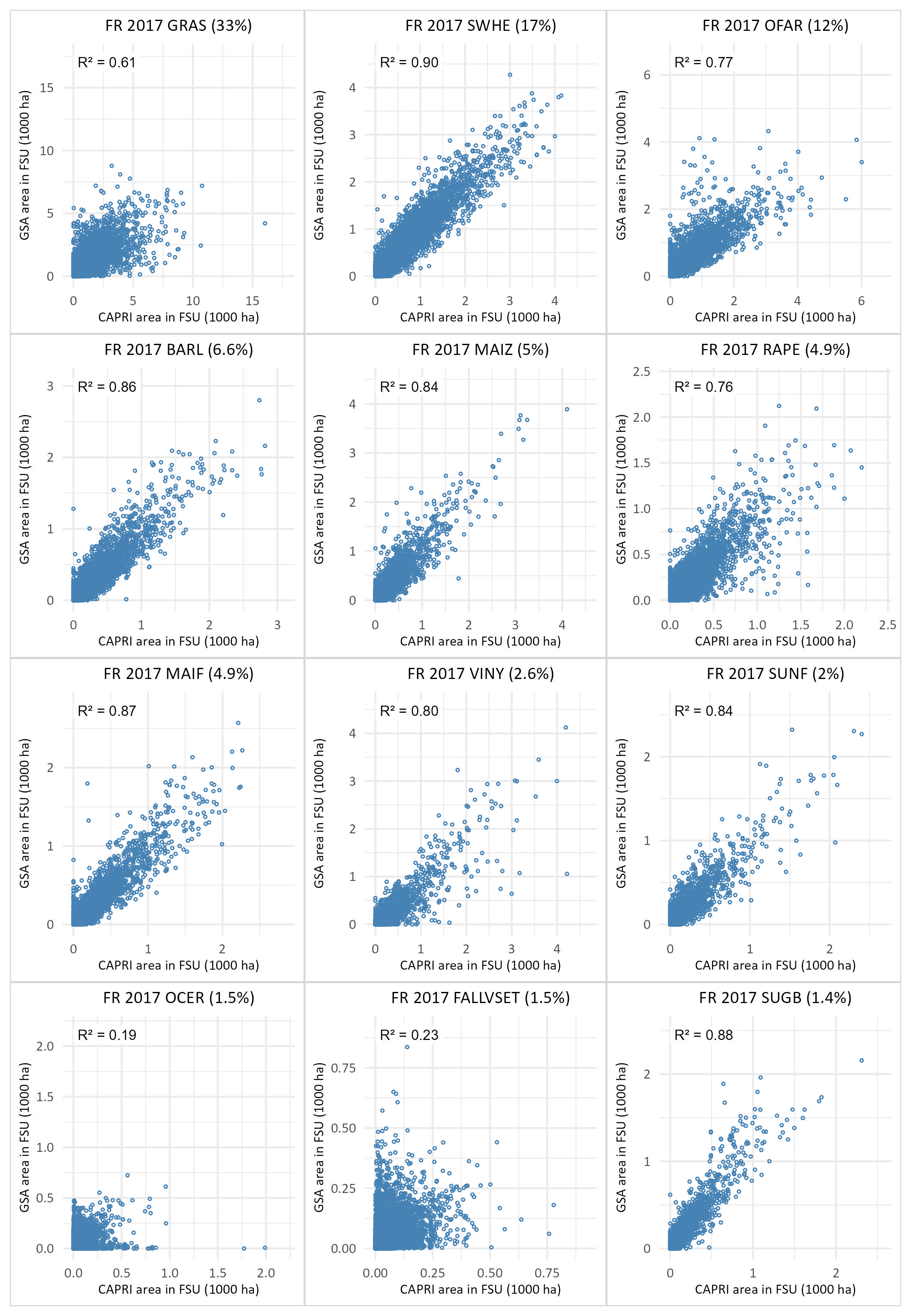


Figure S8: Comparison of the crops with the largest shares in France from the GSA with the same crops from CAPRI by FSU for the year 2017. The meaning of the crop codes can be found in Table 1 of the main paper.

Table S1: Comparison of the CAPRI disaggregation results with GSA data. The share of the matching crop area is calculated according equations 2 - 5 in the main paper for 5 regions (AT=Austria, BEFL = Flanders, BEWA = Wallonia) for the years 2010 and 2017 (data for 2010 was available only for Flanders). The meaning of the crop codes can be found in Table 1 of the main paper.

|  | AT | | | BEFL | | | | | | BEWA | | |
| --- | --- | --- | --- | --- | --- | --- | --- | --- | --- | --- | --- | --- |
|  | 2017 | | | 2010 | | | 2017 | | | 2017 | | |
| **Crop_Type** | CAPRI (kha) | GSA (kha) | MATCH AREA (%) | CAPRI (kha) | GSA (kha) | MATCH AREA (%) | CAPRI (kha) | GSA (kha) | MATCH AREA (%) | CAPRI (kha) | GSA (kha) | MATCH AREA (%) |
| **All Crops** | 2752 | 3201 | **77** | 608 | 667 | **75** | 584 | 663 | **77** | 700 | 769 | **74** |
| **GRAS** | 1349 | 1815 | 77 | 169 | 168 | 76 | 160 | 243 | 87 | 310 | 374 | 74 |
| **SWHE** | 274 | 274 | 84 | 73 | 76 | 85 | 66 | 65 | 82 | 134 | 141 | 87 |
| **OFAR** | 136 | 143 | 85 | 0 | 98 | 0 | 56 | 16 | 26 | 38 | 9 | 22 |
| **BARL** | 139 | 139 | 84 | 11 | 13 | 85 | 16 | 16 | 80 | 30 | 32 | 77 |
| **MAIF** | 85 | 82 | 81 | 120 | 121 | 87 | 115 | 126 | 90 | 48 | 54 | 85 |
| **MAIZ** | 210 | 211 | 84 | 63 | 62 | 83 | 37 | 47 | 89 | 4 | 5 | 67 |
| **RAPE** | 41 | 40 | 73 | 1 | 1 | 49 | 1 | 1 | 38 | 10 | 11 | 72 |
| **FALLVSET** | 73 | 57 | 59 | 3 | 4 | 41 | 1 | 1 | 26 | 3 | 2 | 46 |
| **VINY** | 48 | 41 | 68 | 1 | 0 | 2 | 1 | 0 | 9 | 1 | 0 | 2 |
| **SUNF** | 22 | 22 | 74 | 0 | 0 | 0 | 0 | 0 | 0 | 0 | 0 | 0 |
| **SUGB** | 43 | 43 | 82 | 25 | 21 | 74 | 16 | 21 | 88 | 30 | 44 | 93 |
| **OCER** | 67 | 67 | 52 | 9 | 4 | 42 | 7 | 2 | 27 | 5 | 7 | 40 |
| **POTA** | 23 | 23 | 56 | 47 | 44 | 78 | 50 | 55 | 83 | 37 | 42 | 86 |
| **DWHE** | 23 | 23 | 73 |  |  |  |  |  |  |  |  |  |
| **PULS** | 23 | 25 | 71 | 1 | 3 | 25 | 1 | 3 | 43 | 3 | 1 | 12 |
| **TOMAOVEG** | 18 | 16 | 44 | 48 | 28 | 51 | 33 | 33 | 73 | 25 | 25 | 72 |
| **OATS** | 26 | 26 | 77 | 1 | 0 | 0 | 1 | 1 | 57 | 4 | 4 | 61 |
| **SOYA** | 65 | 64 | 71 | 0 | 0 | 0 | 0 | 0 | 0 | 0 | 0 | 0 |
| **RYEM** | 37 | 37 | 74 | 0 | 0 | 32 | 0 | 0 | 31 | 0 | 0 | 24 |
| **APPLOFRU** | 9 | 14 | 49 | 14 | 15 | 41 | 10 | 16 | 47 | 7 | 2 | 18 |
| **OCRONECR** | 8 | 4 | 27 | 1 | 1 | 15 | 1 | 1 | 12 | 0 | 0 | 11 |
| **TEXT** | 1 | 2 | 20 | 9 | 3 | 27 | 8 | 4 | 34 | 7 | 13 | 85 |
| **OOIL** | 29 | 29 | 58 | 0 | 0 | 13 | 0 | 0 | 19 | 0 | 0 | 5 |
| **OIND** | 0 | 5 | 96 | 1 | 1 | 10 | 0 | 1 | 15 | 0 | 0 | 12 |
| **ROOF** | 0 | 0 | 16 | 5 | 3 | 50 | 4 | 4 | 77 | 1 | 1 | 43 |
| **NURS** | 3 | 1 | 11 | 1 | 0 | 13 | 1 | 4 | 75 | 1 | 1 | 35 |
| **FLOW** | 0 | 0 | 5 | 4 | 2 | 27 | 1 | 1 | 53 | 0 | 0 | 2 |
| **LOLIV** |  |  |  |  |  |  |  |  |  |  |  |  |
| **PARI** |  |  |  |  |  |  |  |  |  |  |  |  |
| **TOBA** | 0 | 0 | 0 | 1 | 0 | 1 | 0 | 0 | 0 | 0 | 0 | 0 |
| **CITR** |  |  |  |  |  |  |  |  |  |  |  |  |
| **UAAR** | 2752 | 3201 | 84 | 608 | 667 | 90 | 584 | 663 | 91 | 700 | 769 | 87 |

Table S2: Comparison of the CAPRI disaggregation results with GSA data. The share of the matching crop area is calculated according equations 2 - 5 in the main paper for 5 regions (DK = Denmark, FR = France) for the years 2010 and 2017 (data for 2010 was available only for Denmark). The meaning of the crop codes can be found in Table 1 of the main paper.

|  | DK | | | | | | FR | | |
| --- | --- | --- | --- | --- | --- | --- | --- | --- | --- |
|  | 2010 | | | 2017 | | | 2017 | | |
| **Crop_Type** | CAPRI (kha) | GSA (kha) | MATCH AREA (%) | CAPRI (kha) | GSA (kha) | MATCH AREA (%) | CAPRI (kha) | GSA (kha) | MATCH AREA (%) |
| **All Crops** | 2781 | 2658 | **82** | 2751 | 2554 | **76** | 28330 | 27872 | **75** |
| **GRAS** | 203 | 202 | 76 | 223 | 206 | 76 | 9315 | 8935 | 71 |
| **SWHE** | 826 | 753 | 84 | 599 | 583 | 85 | 4864 | 4976 | 87 |
| **OFAR** | 388 | 401 | 91 | 324 | 311 | 85 | 3470 | 3222 | 75 |
| **BARL** | 608 | 566 | 87 | 679 | 661 | 88 | 1878 | 1905 | 82 |
| **MAIF** | 192 | 173 | 81 | 172 | 167 | 81 | 1378 | 1283 | 78 |
| **MAIZ** | 9 | 10 | 56 | 5 | 6 | 33 | 1429 | 1557 | 81 |
| **RAPE** | 176 | 164 | 82 | 178 | 176 | 80 | 1385 | 1405 | 78 |
| **FALLVSET** | 60 | 32 | 41 | 212 | 33 | 15 | 428 | 443 | 55 |
| **VINY** | 0 | 0 | 0 | 0 | 0 | 0 | 735 | 578 | 67 |
| **SUNF** | 0 | 0 | 0 | 0 | 0 | 0 | 578 | 584 | 75 |
| **SUGB** | 45 | 39 | 80 | 35 | 34 | 83 | 391 | 500 | 89 |
| **OCER** | 41 | 45 | 79 | 9 | 16 | 59 | 431 | 429 | 53 |
| **POTA** | 44 | 38 | 69 | 50 | 49 | 70 | 188 | 190 | 74 |
| **DWHE** |  |  |  |  |  |  | 368 | 371 | 73 |
| **PULS** | 14 | 10 | 51 | 22 | 21 | 36 | 348 | 368 | 65 |
| **TOMAOVEG** | 12 | 11 | 59 | 12 | 13 | 57 | 254 | 212 | 46 |
| **OATS** | 53 | 42 | 70 | 66 | 58 | 68 | 190 | 190 | 52 |
| **SOYA** | 0 | 0 | 0 | 0 | 0 | 0 | 141 | 141 | 61 |
| **RYEM** | 56 | 52 | 71 | 113 | 111 | 64 | 24 | 24 | 32 |
| **APPLOFRU** | 4 | 6 | 80 | 4 | 4 | 56 | 157 | 148 | 33 |
| **OCRONECR** | 22 | 110 | 96 | 30 | 102 | 93 | 54 | 142 | 33 |
| **TEXT** | 4 | 0 | 2 | 4 | 1 | 3 | 113 | 117 | 26 |
| **OOIL** | 14 | 1 | 1 | 4 | 0 | 2 | 47 | 34 | 25 |
| **OIND** | 0 | 1 | 0 | 0 | 1 | 0 | 82 | 57 | 39 |
| **ROOF** | 6 | 0 | 0 | 4 | 0 | 0 | 25 | 18 | 35 |
| **NURS** | 3 | 1 | 35 | 4 | 2 | 20 | 8 | 10 | 25 |
| **FLOW** | 2 | 1 | 7 | 2 | 0 | 7 | 7 | 0 | 0 |
| **LOLIV** |  |  |  |  |  |  | 17 | 13 | 50 |
| **PARI** |  |  |  |  |  |  | 17 | 15 | 40 |
| **TOBA** |  |  |  |  |  |  | 3 | 3 | 43 |
| **CITR** |  |  |  |  |  |  | 4 | 2 | 17 |
| **UAAR** | 2781 | 2658 | 90 | 2751 | 2554 | 89 | 28330 | 27872 | 85 |

**Comparison of CAPRI mineral fertilizer and manure N time series with other sources**

In addition to the comparison of CAPRI N data with other sources for a **single year** at country level (Figure 9 in the main text), we also compared at EU (+UK) level the **time series** of CAPRI N input from mineral fertilizer and manure with data from (i) statistical database of FAO (FAOSTAT)^13,14^, (ii) data reported in the National Inventory Reports (NIR) of countries to the UNFCCC^15^ and (iii) to the HaNi (History of anthropogenic Nitrogen inputs) data set of Tian et al.^16,17^. The latter includes annual rates of synthetic N fertilizer, manure application/deposition, and atmospheric N deposition on cropland, pasture, and rangeland at a spatial resolution of 5 arc minutes globally.

The results of the comparison of these data for N mineral fertilizer in the soil and N in manure as applied to the soil by the farmer and manure left on pasture by grazing animals in 2000, 2005, 2010 and 2018 is shown in Figure S9 a and b. The overall level of agreement between the different sources is good. However, in contrast to the data from FAOSTAT, NIR and HaNi, the CAPRI N in manure applied to the soil and from grazing animals does not show a steadily decreasing trend between 2000 and 2018 in the EU + UK area (Figure S9 b).


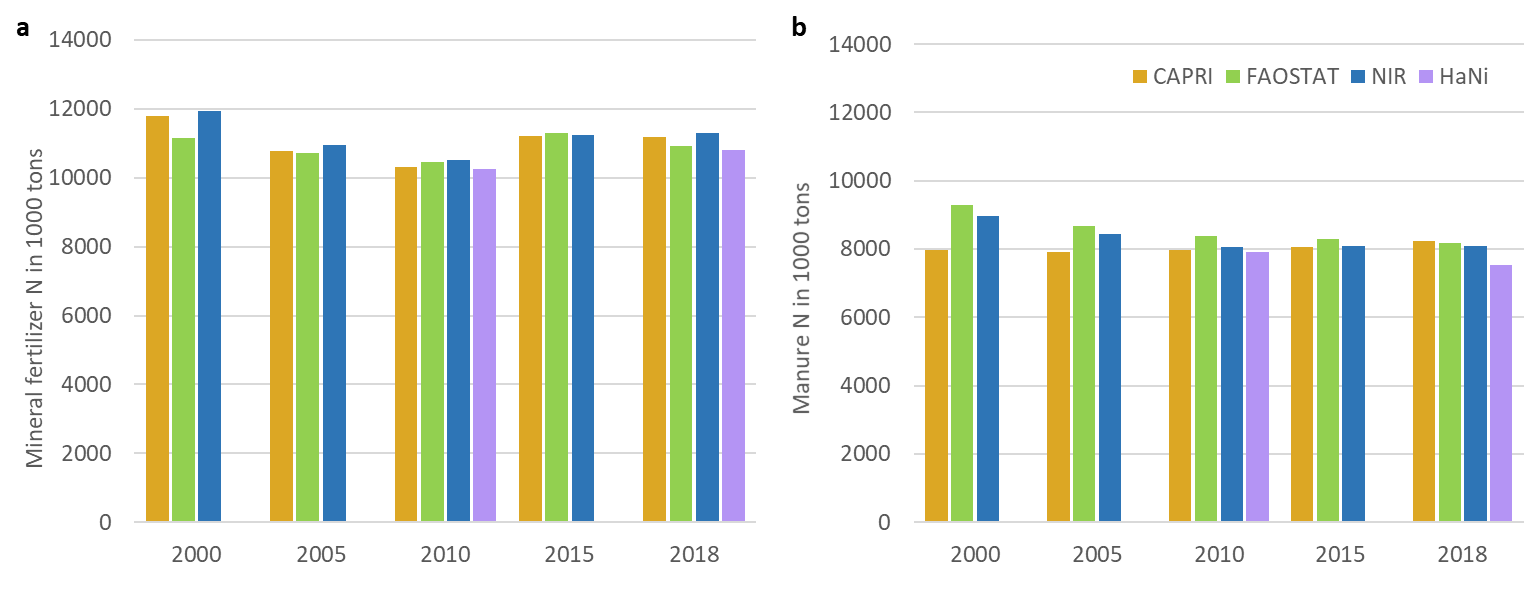


Figure S9: Comparison of N mineral fertilizer input to the soil (a) as well as N in manure applied to the soil by the farmer and manure left on pasture by grazing animals (b) in 2000, 2005, 2010 and 2018 in the EU (excl. Croatia) and UK from the CAPRI model, FAOSTAT^14^, the National Inventory Reports (NIR) of countries to the UNFCCC^15^ and from the HaNi (History of anthropogenic Nitrogen inputs) data set of Tian et al.^16,17^. Note: N losses during manure management before application are excluded here.

The reasons for this discrepancy can be explained by various factors such as the differences in the livestock data, estimation of excretion, emissions from manure management systems or differences in a combination of these factors. One main difference regarding livestock data is related to the accounting. CAPRI applies a different approach compared to most national statistics (Eurostat and FAOSTAT). CAPRI livestock numbers refer to the number of animals produced per year, which also takes slaughter statistics into account, whereas livestock numbers given by Eurostat^18^ and FAOSTAT^19^ refer to the animals present on a reference day within a year.

As shown in Figure S10, livestock numbers based on the accounting of FAOSTAT^19^ decreased between 2000 and 2018 while the meat production from animals slaughtered in the EU showed an increasing trend (12.7%) in the same period, indicating intensification (either more animals raised annually per location or increased weight per animal). In fact, the meat yield per animal increased by 14% for cattle, 15% for poultry and 5% for pigs between 2000 and 2018^19^. For the reasons mentioned above, CAPRI livestock production numbers are around 50% larger compared to the values reported by FAOSTAT. Contrary to the trend in FAOSTAT data, CAPRI livestock production numbers show a slight decrease between 2000 and 2009 but increased again from 2010 onwards. FAOSTAT meat production also reflects increasing intensities captured by CAPRI, although with a much less pronounced pattern. CAPRI as well as FAOSTAT quantities of N from manure (application and grazing) follow the trend of the respective livestock data.


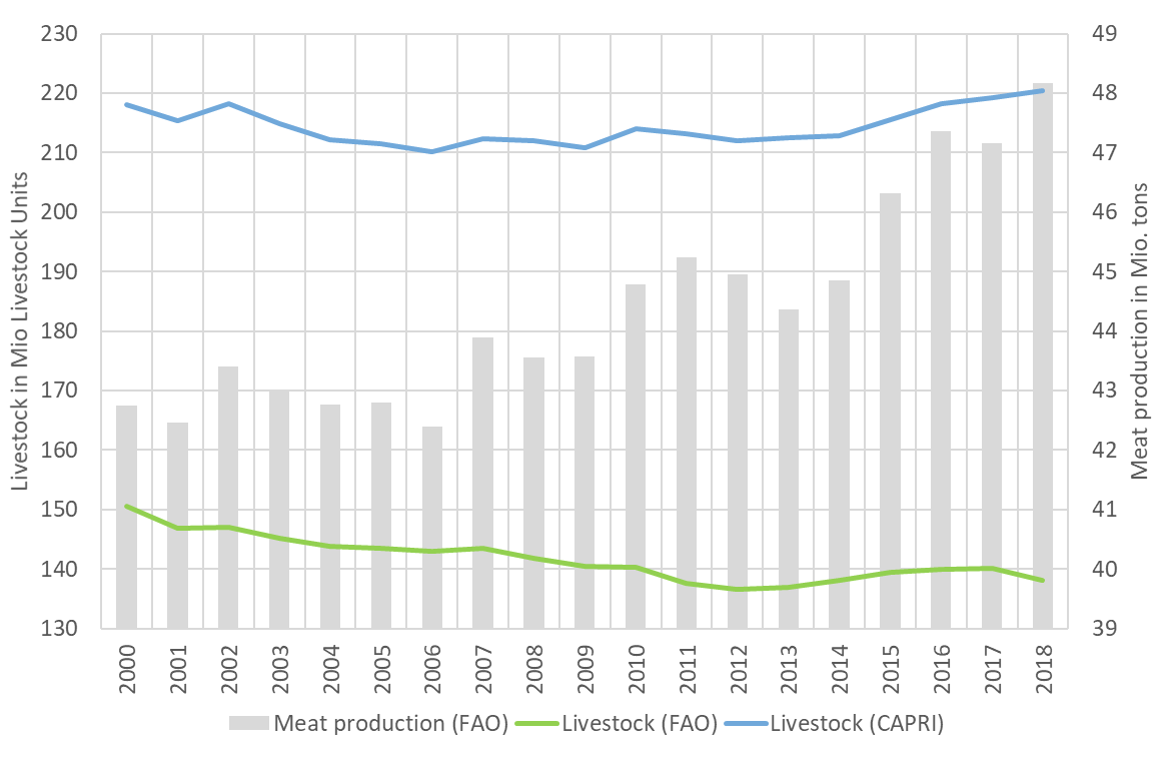


Figure S10: Livestock and meat production in the EU27 and UK from FAOSTAT^19^ and CAPRI. Meat production relates to animals slaughtered within national boundaries, irrespective of their origin^20^. However, most of the animals slaughtered in the EU were also raised in the EU. In 2017-2021, cross-border transports of live animals in the EU^21^ consisted of intra-EU movements of 86%, 13.5% of exports outside the EU and 0.5% of imports from non-EU countries.

There are also some discrepancies between the other data sets. Although the HaNi data set^16^ is based on FAOSTAT data of N in manure, the reason for the differences in the quantities between FAOSTAT and HaNi for the year 2010 and 2018 is not known. The livestock statistics underlying the calculations in the NIR to UNFCCC were not available. However, the temporal trend for N in manure in the NIR follows the FAOSTAT trend, although the quantities are slightly lower.

### References

1. Britz, W. Automated model linkages: the example of CAPRI. Agrarwirtschaft 57, 363–367. DOI: 10.22004/ag.econ.97707 (2008).
2. JRC. CAPRI. Common Agricultural Policy Regional Impact Analysis. https://web.jrc.ec.europa.eu/policy-model-inventory/explore/models/model-capri/ [Accessed: 22-September-2025].
3. Leip, A., Britz, W., Weiss, F. & De Vries, W. Farm, land, and soil nitrogen budgets for agriculture in Europe calculated with CAPRI. Environmental Pollution 159, 3243–3253, https://doi.org/10.1016/j.envpol.2011.01.040 (2011).
4. Weiss, F. & Leip, A. Greenhouse gas emissions from the EU livestock sector: A life cycle assessment carried out with the CAPRI model. Agriculture, Ecosystems & Environment 149, 124–134, https://doi.org/10.1016/j.agee.2011.12.015 (2012).
5. Henning, C. & Witzke, P. Economic and Environmental Impacts of the Green Deal on the Agricultural Economy: A Simulation Study of the Impact of the F2F-Strategy on Production, Trade, Welfare and the Environment Based on the CAPRI-Model. Available at https://vakbladvoedingsindustrie.nl/storage/app/media/Rapporten/RAPPORTEN%202021/VOE-2021-OKT-KIEL.pdf (2021).
6. Wąs, A., Zawalińska, K. & Britz, W. Impact of ‘greening’ the Common Agricultural Policy: Evidence from selected countries based on CAPRI model, International Congress, August 26-29, 2014, Ljubljana, Slovenia, European Association of Agricultural Economists. DOI: 10.22004/ag.econ.186374 (2014).
7. Blanco, M., Witzke, P., Pérez Dominguez, I., Salputra, G. & Martinez, P. Extension of the CAPRI Model with an Irrigation Sub-Module. EUR 27737 EN; Doi:10.2791/319578. (2015).
8. Agrarmarkt Austria. INVEKOS Schläge Österreich 2017. Available at: <https://inspire.lfrz.gv.at/009501/ds/inspire_schlaege_2017_polygon.gpkg.zip> [Accessed: 24-July-2022].
9. Landbrugsstyrelsen. LandbrugsGIS, Marker. Available at: <https://landbrugsgeodata.fvm.dk/> (Files: Marker_2010.zip, Marker_2017.zip) [Accessed: 1-July-2020].
10. Digital Vlaaanderen, Departement Landbouw en Visserij. Landbouwgebruikspercelen. Available at: <https://metadata.vlaanderen.be/srv/dut/catalog.search#/metadata/47c5540f-bf7c-45fc-9a74-8e60547cde82> [Accessed: 15-July-2022].
11. Service public de Wallonie (SPW) - INSPIRE - Parcellaire agricole anonyme 2017 en Wallonie (BE), Available at: <https://geoportail.wallonie.be/catalogue/bae58e43-d9cb-4996-93d0-61e722e61499.html#tabs-3> (Jaar 2017, ESRI Shape) [Accessed: 21-June-2023].
12. Institut national de l'information géographique et forestière (IGN-F), Registre Parcellaire Graphique, Available at: <https://geoservices.ign.fr/rpg> (RPG 2017, RPG France métropolitaine) [Accessed: 1-July-2020].
13. FAOSTAT. Fertilizers by Nutrient. Available at: https://www.fao.org/faostat/en/#data/RFN (Data selection: EU countries + UK, Agricultural Use, Nutrient Nitrogen N total, Year 2018) [Accessed: 15-May-2024].
14. FAOSTAT. Livestock Manure. Available at: https://www.fao.org/faostat/en/#data/EMN (Data selection: EU countries + UK, Agricultural Use, Manure applied to soils N content and Manure left on pasture N content, All animals, Year 2018) [Accessed: 15-May-2024]
15. United Nations Framework Convention on Climate Change (UNFCCC). National Inventory Reports, Common reporting format (CRF) tables. Available at: <https://unfccc.int/process-and-meetings/transparency-and-reporting/reporting-and-review/reporting-and-review-under-the-convention/greenhouse-gas-inventories-annex-i-parties/submissions/national-inventory-submissions-2022> (Data: CRF Table3.D, Inorganic N fertilizers, Animal manure applied to soils, Urine and Dung depositied by grazing animals) [Accessed: 16-March-2023].
16. Tian, H. et al. HaNi: A Historical dataset of Anthropogenic Nitrogen Inputs to the terrestrial biosphere (1860–2019). PANGAEA, <https://doi.org/10.1594/PANGAEA.942069> (2022)
17. Tian, H. et al. History of anthropogenic Nitrogen inputs (HaNi) to the terrestrial biosphere: a 5 arcmin resolution annual dataset from 1860 to 2019. Earth Syst. Sci. Data 14, 4551–4568, https://doi.org/10.5194/essd-14-4551-2022 (2022).
18. Eurostat. Farm structure. https://ec.europa.eu/eurostat/cache/metadata/en/ef_sims.htm [Accessed: 15-June-2025]
19. FAOSTAT. Crops and livestock products. Available at: https://www.fao.org/faostat/en/#data/QCL (Data selection: COUNTRIES: EU countries + UK, ELEMENTS:Production Quantity, ITEMS:Livestock primary, Meat -total- and ELEMENTS:Stocks, ITMES: Live Animals, Cattle and Buffaloes, Chickens, Sheep/Goats, Swine/Pigs, YEARS: 2000 - 2018) [Accessed: 15-May-2024]
20. FAOSTAT. QCL methodology, <https://files-faostat.fao.org/production/QCL/QCL_methodology_e.pdf> [Accessed: 15-May-2024]
21. European Court of Auditors, Transport of live animals in the EU: challenges and opportunities, Review 03, https://www.eca.europa.eu/Lists/ECADocuments/RV-2023-03/RV-2023-03_EN.pdf (2023).
